# Molecular Docking and QSAR Studies as Computational Tools Exploring the Rescue Ability of F508del CFTR Correctors

**DOI:** 10.3390/ijms21218084

**Published:** 2020-10-29

**Authors:** Giada Righetti, Monica Casale, Nara Liessi, Bruno Tasso, Annalisa Salis, Michele Tonelli, Enrico Millo, Nicoletta Pedemonte, Paola Fossa, Elena Cichero

**Affiliations:** 1 Department of Pharmacy, Section of Medicinal Chemistry, School of Medical and Pharmaceutical Sciences, University of Genoa, Viale Benedetto XV, 3, 16132 Genoa, Italy; righetti@difar.unige.it (G.R.); tasso@difar.unige.it (B.T.); tonelli@difar.unige.it (M.T.); fossa@difar.unige.it (P.F.); 2 Department of Pharmacy, Section of Chemistry and Food and Pharmaceutical Technologies, University of Genoa, Viale Cembrano, 4, 16148 Genoa, Italy; casale@difar.unige.it; 3 Department of Experimental Medicine, Section of Biochemistry, University of Genoa, Viale Benedetto XV 1, 16132 Genoa, Italy; liessi_nara@libero.it; 4 Center of Excellence for Biomedical Research (CEBR), University of Genoa, Viale Benedetto XV 9, 16132 Genoa, Italy; annalisa.salis@unige.it; 5 UOC Genetica Medica, IRCCS Istituto Giannina Gaslini, 16047 Genova, Italy; nicoletta.pedemonte@unige.it

**Keywords:** CFTR, corrector, QSAR, docking, cystic fibrosis, VX-809

## Abstract

Cystic fibrosis (CF) is the autosomal recessive disorder most recurrent in Caucasian populations. Different mutations involving the cystic fibrosis transmembrane regulator protein (CFTR) gene, which encodes the CFTR channel, are involved in CF. A number of life-prolonging therapies have been conceived and deeply investigated to combat this disease. Among them, the administration of the so-called CFTR modulators, such as correctors and potentiators, have led to quite beneficial effects. Recently, based on QSAR (quantitative structure activity relationship) studies, we reported the rational design and synthesis of compound **2**, an aminoarylthiazole-VX-809 hybrid derivative exhibiting promising F508del-CFTR corrector ability. Herein, we explored the docking mode of the prototype VX-809 as well as of the aforementioned correctors in order to derive useful guidelines for the rational design of further analogues. In addition, we refined our previous QSAR analysis taking into account our first series of in-house hybrids. This allowed us to optimize the QSAR model based on the chemical structure and the potency profile of hybrids as F508del-CFTR correctors, identifying novel molecular descriptors explaining the SAR of the dataset. This study is expected to speed up the discovery process of novel potent CFTR modulators.

## 1. Introduction

Cystic fibrosis transmembrane conductance regulator (CFTR) is a glycoprotein encoded by the CFTR consisting of 6 domains: two transmembrane domains (MSD1 and MSD2), two nucleotide binding domains (NBD1 and NBD2), a regulatory “R” region, and a PDZ interacting motif [1,2,3]. CFTR is a member of the ABC transporter ATPase family and allows for the transmembrane flow of chloride ions (Cl^−^) down the electrochemical gradient [1]. Such anion flow is critical for proper water balance between the extracellular and intracellular space of epithelial cells in the gastrointestinal tract, pulmonary tract, and ducts lining organs such as the pancreas, testes, and sweat glands [4,5,6]. Without proper water flow, the mucus lining of these tracts becomes dehydrated and accumulates in these areas giving rise to bacterial infections, especially in the lungs [7,8].

A variety of mutations lead to defective function of this channel, which lead to the pathological condition known as cystic fibrosis (CF) [1]. Deletion of phenylalanine 508 (F508del-CFTR) is the most common deletion related to CFTR [9,10] and results in the improper folding of the protein. This event causes protein degradation before it can reach the plasma membrane of epithelial cells [9,10,11,12].

The F508 residue is located on the surface of NBD1 and is important to properly guide inter-domain assembly with NBD2 and MSD2 and then trafficking the protein to the plasma membrane [6,7,13]. As a consequence, most of the F508del-CFTRs are targeted for degradation in the endoplasmic reticulum. Conversely, other mutations may result in a protein that is translocated to the membrane but in reduced number or functionality [14,15].

The primary defects caused by CFTR mutations can be treated with drug-like small molecules, known as “CFTR modulators”, targeting specific defects caused by mutations in the CFTR gene. They are classified into five main groups including read-through agents, correctors, potentiators, stabilizers and amplifiers [11].

In particular, main efforts revolve around the discovery of compounds that correct at least CFTR misfolding and ER retention as well as defective channel gating, which are considered the two major underlying problems in CF [16,17].

It is believed that the folding and the channel activity defect of F508del can be addressed by means of modulators named correctors and potentiators, respectively. Correctors improve the trafficking of mutant CFTR to the plasma membrane; meanwhile, potentiators should bind to F508del-CFTR at the cell surface and increase chloride channel gating.

Over the last years, several efforts have been taken to identify new promising chemical entities endowed with a F508del-CFTR corrector and/or potentiator behavior, including high-throughput screening strategies (HTS) [17,18,19,20,21,22].

**Figure 1:**
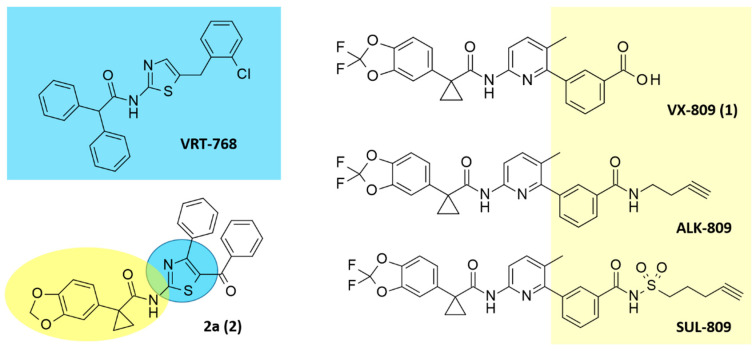
Chemical structure of VRT-768 as hit compound in the search of F508del-CFTR (cystic fibrosis transmembrane regulator protein) correctors. The optimized lead VX-809 is shown together with the highly related analogues ALK-809 and SUL-809. The hybrid **2** is depicted, featuring the main thiazole core of the previous VRT-768 (in cyan) as well as key features of VX-809 (in yellow).

In this context, Vertex Pharmaceuticals discovered cycloalkyl carboxamide F508del-CFTR correctors, firstly identifying a thiazole-containing compound (VRT-768; corrector EC_50_ = 16µM–pEC_50_ = 4.80 M) as a prototype (Figure 1). This compound was further optimized towards the well-known corrector VX-809 (Lumacaftor; corrector EC_50_ = 2.6 µM–pEC_50_ = 5.59 M), bearing a pyrimidine ring as bioisosteres of the previous thiazole nucleus (Figure 1) [23].

Then, the same authors also investigated dual-acting derivatives bearing a cyanoquinoline (CQ) core linked to an aryl amide moiety (by a variable tether), displaying independent corrector and potentiator activities [24]. More recently, a screening campaign reported by other research groups enlightened a number of tetrahydropyridopyrimidines, properly decorated with aromatic substituents and basic moieties, as novel promising CFTR correctors [25].

Despite this, VX-809 (Lumacaftor) and the structurally related analogue VX-661 (Tezacaftor) are the only approved correctors for clinical use, administered in combination with the potentiator VX-770 (Ivacaftor). Since the treatment is modestly effective [26], there is an urgent need to identify other more effective compounds to be translated into the clinic. Thus, while the combination therapy Lumacaftor-Ivacaftor reached the market, other triple-combinations are under evaluation, such as Elexacaftor (VX-445)-Tezacaftor-Ivacaftor [27]. In particular, this triple combination provides potential therapy for many patients who had previously been excluded from CFTR modulation treatment. The efficacy demonstrated in clinical trials exceeds currently available therapies even though it is believed that at least 10% of subjects do not respond to this combination.

In the search for new CFTR modulators, we designed and synthesized a number of aminoarylthiazoles (AATs) able to act as modulators of F508del-CFTR [28,29].

Successively, novel derivatives conceived, including the AAT core, merged with the benzodioxole carboxamide moiety featured by VX-809 (namely hybrids) and were designed [30].

All the compounds were active as correctors, with compound **2** the most effective of the series (pEC_50_ = 7.06; Figure 1).

Beyond this ligand-based strategy, the rational design of novel F508del-CFTR correctors appears to be complicated by the absence of clear information about the mechanism of action supporting the VX-809 rescue ability. Several studies deeply investigated in silico the putative binding mode of VX-809 taking into account different portions of the CFTR channel, suggesting a multiple site-targeting behavior.

Data reported in the literature showed VX-809 directly targeting the full-length F508del-CFTR protein, with a possible binding site at the NBD1-ICL4 interface [31,32]. The same docking studies suggest that VX-809 may bind to F508del-CFTR at NBD1 [33].

Accordingly, recent click chemistry-based approaches combined with biological assays confirmed a direct binding of VX-809 and its alkyne-congeners ALK-809 and SUL-809 on CFTR (Figure 1) [34]. Recent approaches moved the discovery of novel F508del correctors exploring the NBD1:NBD2 interface to the MSD:NBD1 one, supporting a fundamental role played by NBD1 at the corrector binding site [18]. In this context Odolczyk reported four correctors identified by virtual screening disrupting keratin8 F508del-CFTR interaction based on their binding to NBD1 [35].

Notably, our research group recently reported surface plasmon resonance (SPR) studies at the murine NBD1 domain of CFTR performed with the corrector VX-809 and molecular docking and dynamics simulations, highlighting a statistically significant correlation between the observed binding capability to F508del-NBD1 and the results of the computational methods. On the whole, these results demonstrated a strong agreement between the theoretical prediction and the experimental SPR-based data, supporting a key role played at the NBD1 level of CFTR by the corrector VX-809 in exerting any CFTR rescue ability [36]. More recent findings explored the docking mode of AAT and VX-809 at the whole CFTR protein, applying a combined SPR and molecular dynamic simulation approach [18].

**Figure 2:**
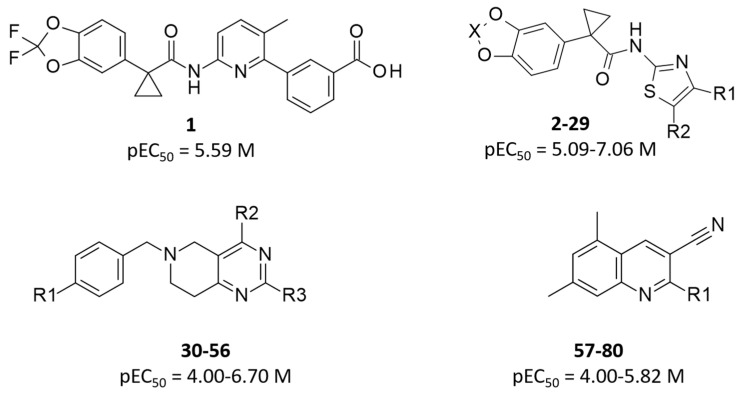
Chemical structure and biological activity range of the investigated F508del-CFTR correctors.

Herein, we continue our molecular modeling studies taking into account both the X-ray crystallographic structure of the NBD1 domain of the human F508del-CFTR mutant as well as an in-house model of the complete CFTR mutant. This kind of approach allowed us to rely on experimental even if partial data (NBD1 domain) and on a modelled but complete structure of the whole biological target (modelled F508del-CFTR). We thought to perform thorough molecular docking simulations with the aim at clarifying the putative binding mode of VX-809 and related analogues, including the in-house series of hybrids, cyanoquinolines and tetrahydropyridopyrimidines (Figure 2).

These computational studies allowed us to deepen the knowledge of the structure–activity relationship around the most recurrent chemotypes so far exploited in the search of novel CFTR modulators, as well as to refine QSAR (quantitative structure activity relationship) analyses including the potent hybrid compounds in the mathematical model, which was generated taking into account the compound conformers selected by docking calculations. This approach will help the optimization process of the already applied rational design protocol, which will be applied to accelerate the design of novel, more promising hybrids.

## 2. Results

### 2.1. Molecular Docking

In this work, we performed molecular docking studies on a library of F508del CFTR correctors (S1-3), featuring variable chemical cores, including the in-house series of VX-809-AAT hybrids as well as modulators reported in the literature. These calculations have been focused on: (i) the human F508del-CFTR NBD1 domain, since several experimental efforts described in the literature strongly remark a key role played by correctors at this domain of the channel [33,34,35,36]; (ii) the whole protein, modelled as F508del-CFTR by means of the available data of the wild-type protein (pdb code: 6MSM) obtained by cryoelectron microscopy [37] and of the mutant NBD1 domain (pdb code: 4WZ6) [38] (see experimental section). The superimposition of the modelled F508del-CFTR onto the 4WZ6 pdb code is shown in S4. In this way, we better explored further putative binding sites involving NBD1 domain.

The obtained scoring functions have been reported in S5–S8. A perspective of these values proved to be in good agreement with the experimental activity, supporting the docking protocol at the two putative binding cavities. As shown in S9, the most promising tetrahydropyridopyrimidines and CQs (featuring pEC_50_ > 5.5) are accompanied by score values at the NBD1 domain lower than –20, while the most effective hybrids (pEC50 > 6.0) exhibited scoring values spanning from −20 to −25. For all the chemotypes herein explored, the poorly active correctors displayed score values higher than −20. Concerning molecular docking at the modelled F508del-CFTR (S10), the same promising hybrids experienced score values spanning from −22 to −30, while the aforementioned tetrahydropyridopyrimidines and CQs showed variable scores spanning from −20 to −30.

We recently explored the putative docking mode of VX-809 and also of the two highly related analogues ALK-809 and SUL-809, chosen as further reference compounds [39]. These two derivatives bring an amide and a sulfonamide function in place of VX-809 carboxylic moiety, lacking the possibility to exhibit a negatively charged group [33].

**Figure 3:**
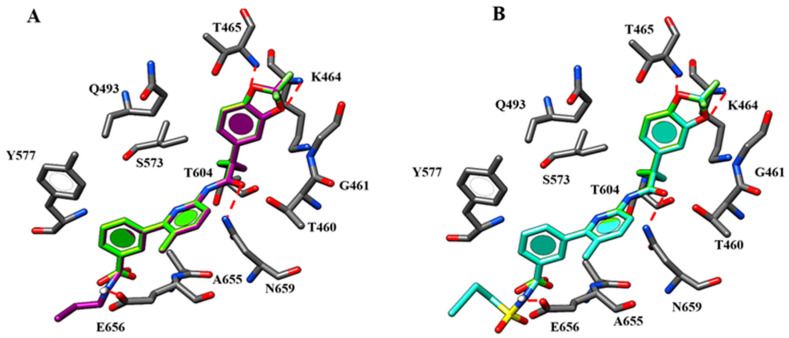
Selected docking poses of ALK-809 (C atom; pink) (**A**) and SUL-809 (C atom; cyan) (**B**) within the CFTR *h*NBD1 domain together with VX-809 (C atom; green). The most important residues are labelled.

Briefly, the two oxygen atoms of the VX-809 benzodioxole core detect two H-bonds with the K464 and T465 while the negatively-charged carboxylic group and the carbamide moiety of the corrector are H-bonded to the backbone of E656 and to N659, respectively (Figure 3).

Concerning ALK-809 and SUL-809, both the two congeners were engaged in one H-bond between the (sulfon)amide moiety and the backbone of E656 being the two oxygen atoms of the benzodioxole near to K464 and T465. In addition, the amide group on the main core of the derivatives was H-bonded to N659 while the aromatic rings and all the substituents were placed in proximity of G461, V603, A655 and N659 residues.

Remarkably, the putative binding site herein identified is in accordance with other computational studies reported in the literature by Odolczyk [35]. Briefly, Odolczyk reported molecular docking and virtual screening strategies identifying four derivatives acting as F508del-CFTR correctors, disclosing two different pockets within the NBD1 domain explaining the corrector ability of the proposed compounds. Based on the higher conformational freedom of the NBD1 domain of F508del-CFTR than WT-NBD1, the authors highlighted the need for molecules targeting the unique conformation of F508del-NBD1 in order to prevent its interaction with housekeeping proteins. Thanks to following molecular dynamic simulations they described the NBD1 pocket 1 and pocket 2, including in particular the first one Q493, N659 and Y577. These theoretical studies were validated by experimental data showing increases in current density comparable to that induced by the reference corrector VX-809 when cells were treated by compounds targeting pocket 1 as well by derivatives binding to pocket 2. The research group conceived their work being aware that data reported in the literature highlighted NBD1 as a key anchoring point for CFTR modulators and that the corrector VRT-325 was reported as a competitive ligand for ATP [40]. These findings depicted the related binding site a plausible even if unwanted way to achieve corrector ability.

**Figure 4:**
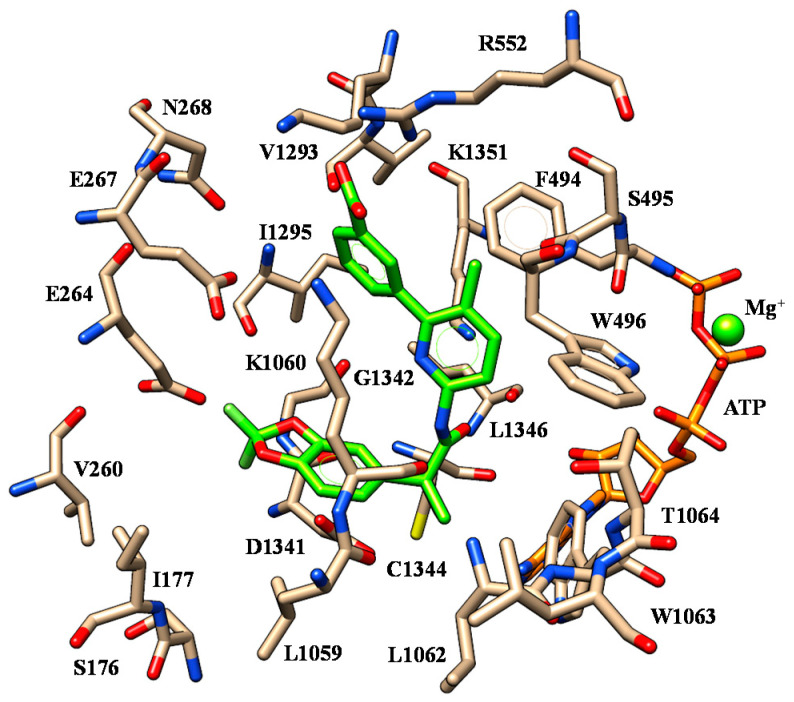
Selected docking pose of VX-809 (C atom; green) within the modelled F508del-CFTR. The most important residues are labelled.

At the mutant protein model, VX-809 was highly stabilized within the protein crevice thanks to polar contacts with R552, K1060 and K1292. As shown in Figure 4, the acid moiety displayed salt-bridges with R552 and K1292 while the two oxygen atoms of the benzodioxole were H-bonded to K1060. 

The folded conformation experienced by the corrector allowed VX-809 to exhibit a further H-bond involving the carboxamide nitrogen atom and K1060. As a consequence, the terminal carboxy-substituted phenyl ring was exposed to F494 and K1060 detecting cation-π contacts and π-π stacking while the pyridine ring was in π-π contact with W496. Finally, the cyclopropyl moiety displayed van der Waals interactions with W1063 and C1344. 

**Figure 5:**
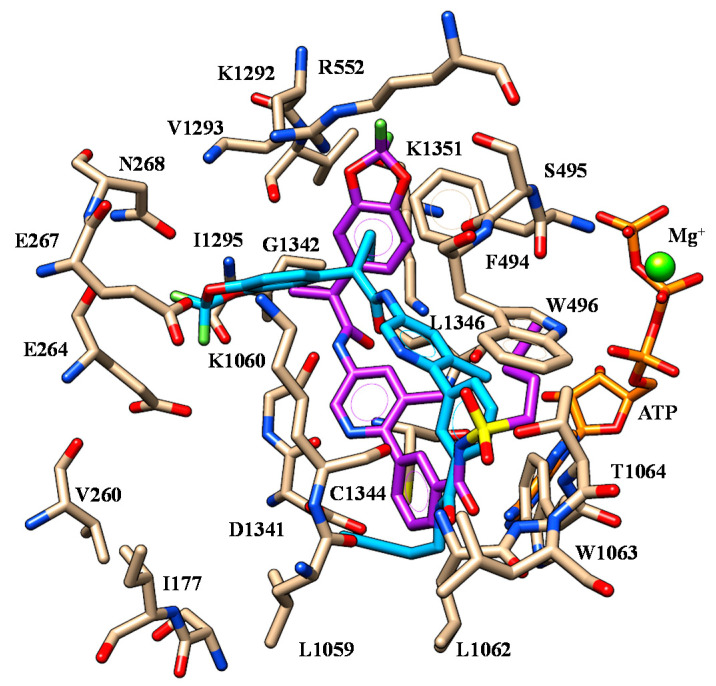
Selected docking poses of ALK-809 (C atom; cyan) and SUL-809 (C atom; purple) within the modelled F508del-CFTR domain. The most important residues are labelled.

The replacement of the carboxy-phenyl ring with the (sulfon) amide substituted phenyl groups led to ALK-809 and SUL-809, endowed by a comparable positioning (Figure 5). In any case, the benzodioxole portion was oriented towards a polar cavity delimited by E264, E267, N268, R552, K1292, V1293 and K1351, detecting one H-bond with I1295 and V1293, respectively.

The central carboxamide moiety of the two correctors showed one H-bond with K1351 while the terminal one was H-bonded to L1062, W1063 (see S11) and K1060, L1062, T1064 (see S11), respectively. This kind of positioning properly arranged the bulkier and lipophilic groups of the modulators in the hydrophobic cavity including W496, L1059, L1063, C1344 and L1346.

Concerning our first series of hybrids obtained merging the AAT main core with the benzodioxole portion of VX-809, derivatives **2**–**4** proved to be the most potent, with **3** and **4** decorated with an ester moiety linked to the position 5 of the thiazole. The promising analogue **2** (pEC_50_ = 7.06) displayed a benzoyl group at the same position (see S1 for the structures).

**Figure 6:**
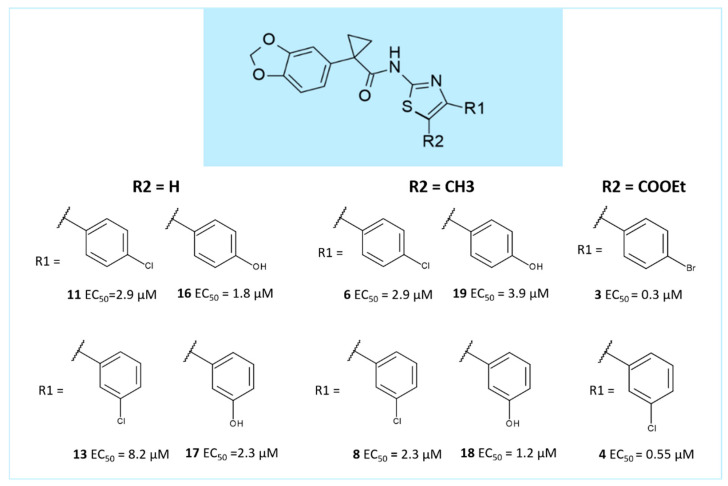
Scheme of the previously disclosed SAR explored for VX-809 and AATs hybrids as F508del-CFTR correctors [30].

Briefly, the structure–activity relationship (SAR) is summarized in Figure 6.

Taking into account 5-unsubstituted AATs, the presence of a *p*-substituted phenyl ring tethered to the position 4 of the main thiazole was privileged, leading to compounds featuring higher potency values if compared to the *m*-substituted analogues.

Conversely, when a methyl group or ester moieties decorate the position 5 of the AAT, the *m*-substitution was more effective. Moreover, similar corrector ability was experienced both by those compounds showing the benzodioxole ring instead of the fluorinate analogue, while a beneficial role was played by concomitant substitutions involving the position 4 and 5 of the thiazole ring [30]. 

**Figure 7:**
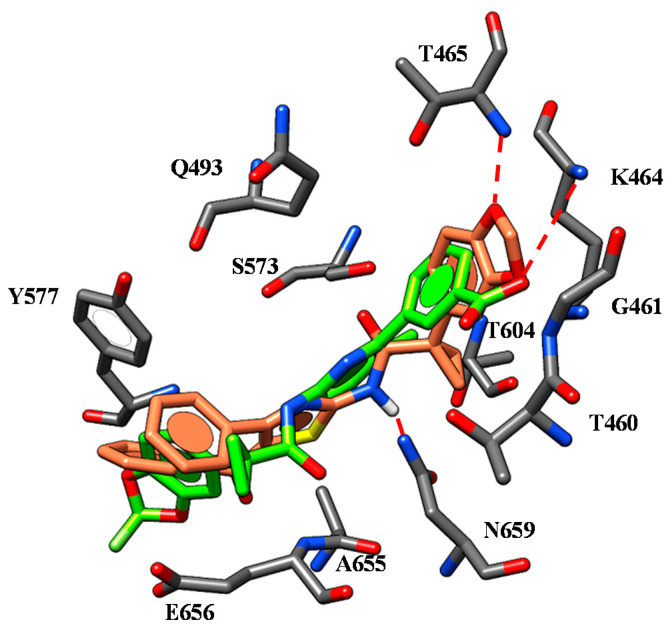
Selected docking mode of **2** (C atom; orange) within the CFTR *h*NBD1 domain in comparison with that of VX-809 (C atom; light green).

Molecular docking calculations on the F508del-CFTR NBD1 domain revealed a highly comparable positioning for all of them, which proved to be in agreement with that previously discussed for VX-809 [39]. Indeed, the carboxamide moiety of the most potent **2** was in proximity of N659 detecting H-bonds, with the two oxygen atoms of the benzodioxole substituent also H-bonded to K464 and T465 (Figure 7).

The benzoyl and the phenyl ring of the hybrid showed π-π stacking with Y577, while the oxygen atom of the benzoyl group interacts with the E656 side chain, as described for the reference corrector. In the case of **3** (pEC_50_ = 6.52) and **4** (pEC_50_ = 6.25), the presence of the quite flexible ester group tethered to the position 5 of the thiazole core allowed the correctors to differently occupy the NBD1 domain on the concomitant *m*- or *p*-substituted phenyl ring connected to the position 4 of AAT (S12). Indeed, **3** and **4** were H-bonded to the hydroxyl group of the Y577 sidechain or to the E656 backbone via the oxygen atom of the carbonyl group, respectively.

However, based on the high corrector ability featured by both two correctors, it is thought that H-bonding N659, K464 and T465 represents a mandatory requirement of correctors, while interacting with E656 or Y577 could efficiently stabilize the compound at the protein domain. The most relevant contacts detected for hybrids at the NBD1 domain are listed in Table S13.

**Figure 8:**
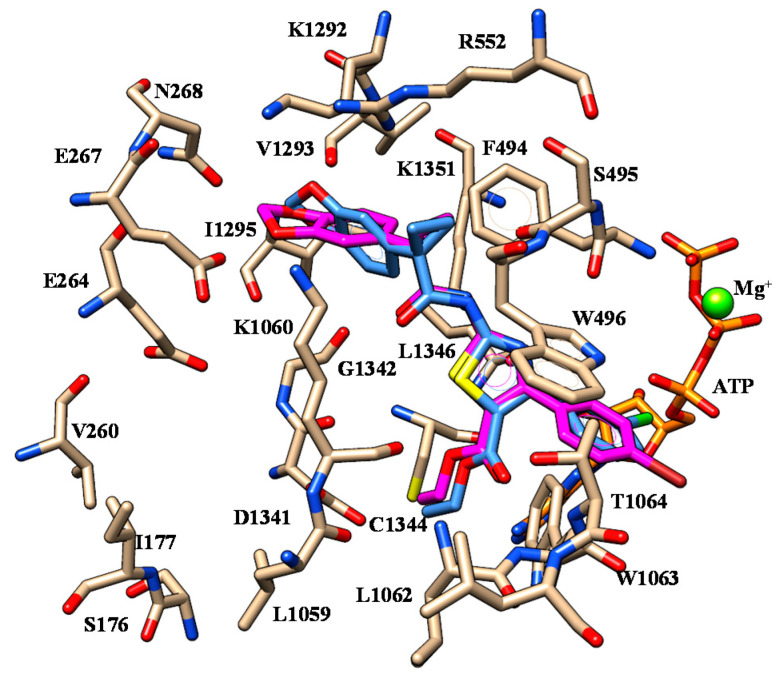
Selected docking mode of **3** (C atom; magenta) and **4** (C atom; light blue) within the modelled F508del-CFTR. The most important residues are labelled.

Deepening molecular docking calculations involving the F508del-CFTR model enlightened key H-bonds between: (i) one oxygen atom of the benzodioxole group and K1060, (ii) the nitrogen atom of the carboxamide moiety and S495, (iii) the ester oxygen atom and W1063 (see Figure 8).

While the benzodioxole group experienced cation-π contacts and π-π stacking with R552 and F494, respectively, the phenyl ring at the position 4 of the thiazole showed π-π contacts with W496 and W1063. On the other hand, the ester group was projected towards C1344 and W1063, in agreement with the docking positioning described for the hydrophobic features of ALK-809 and SUL-809 (S14).

**Figure 9:**
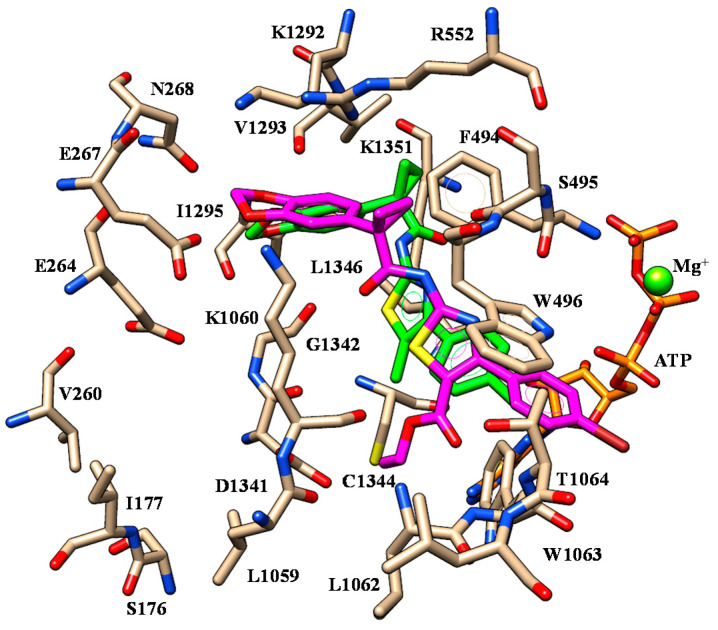
Selected docking mode of **3** (C atom; magenta) and **6** (C atom; green) within the modelled F508del-CFTR. The most important residues are labelled.

The introduction of a smaller group at the position 5 of the thiazole, such as a methyl group instead of the ester or carbonyl moiety, led to compounds **6**-**10** (pEC_50_ = 5.5–6.0) being less potent than the previously cited analogues **2**–**5**. Thus, even if they maintained the overall positioning so far discussed, they displayed different H-bonds involving I1295 and K1351 thanks to the benzodioxole and carboxamide oxygen atoms (Figure 9) while the main π-π stacking and van der Waals contacts were maintained.

Indeed, the benzodioxole and the phenyl ring exhibited lipophilic contacts with F494, V1293 and W496, W1063, respectively. Notably, the overall positioning of **6** highly resembles that of ALK-809, but lacks H-bonds with L1062 and W1063 (S15).

**Figure 10:**
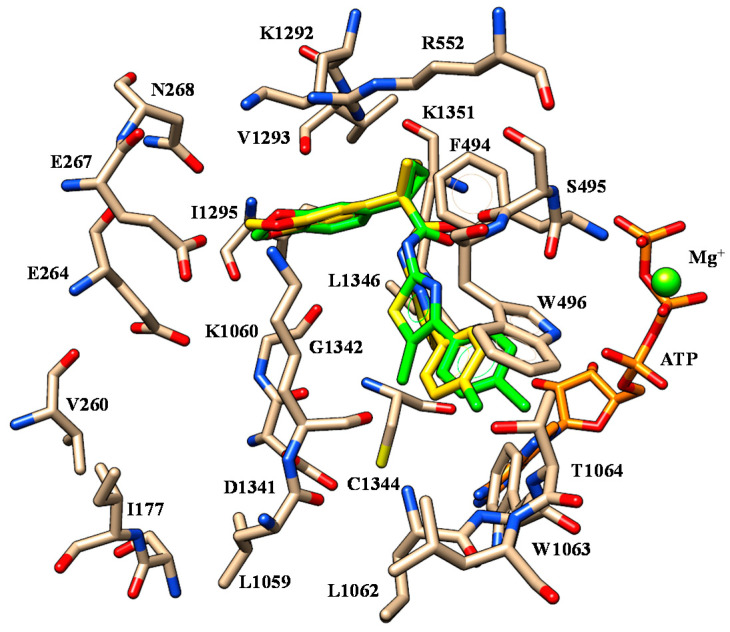
Selected docking mode of **6** (C atom; green) and **11** (C atom; yellow) within the modelled F508del-CFTR. The most important residues are labelled.

Leaving unsubstituted the position 5 of the thiazole core quite impaired the corrector ability of the compounds, as shown for **11**–**13** (pEC_50_ = 5.08–5.53), featuring lower pEC_50_ values than the methyl-substituted analogues **6**–**8** (pEC_50_ = 5.53–5.72). Among them, compound **11** was the most interesting, as it was endowed with the same potency than the analogue **6**. Accordingly, the two hybrids shared comparable positioning (see Figure 10).

Despite this, the presence of the methyl group is thought to be related to the proper conformational and steric requirements which allow the correctors to properly bind the protein cavity, exhibiting the most effective overall shape.

Conversely, the introduction of more lipophilic groups at the *p*-position of the phenyl ring allowed the detection of hydrophobic interactions with W496 and W1063, as shown for **7** compared to **6** (S16). The replacement of halogens with other electron-withdrawing groups lacking hydrophobic properties such as the carboxylic group, led to compounds **20** and **21**, with **21** the most promising (pEC_50_ = 5.53). Interestingly, the related docking pose was endowed with comparable contacts with those previously proposed for VX-809 (S17).

The presence of polar substituents with electron-donor properties such as the hydroxyl function at the meta position of the phenyl ring improved the potency of the modulators, as shown for **17**–**18** (pEC_50_ = 5.64–5.92). In particular, **18** (pEC_50_ = 5.92) was H-bonded to K1060 maintaining all the other contacts discussed for compound **6** (S18).

On the other hand, the series of tetrahydropyridopyrimidine includes compounds **30**–**56** (pEC_50_ = 4.00–6.70) featuring variable substituents onto the positions 2 and 4 of the main core and at the nitrogen atom of the tetrahydropyrido portion (see S2 for the structures).

The presence of an unsubstituted benzyl group in R1 was compatible both with a secondary (**33**, pEC_50_ = 5.51) and a tertiary (**30**, **32**–**34**, pEC_50_ = 5.55–5.82) amine group directly tethered as R2 to position 4 of the tetrahydropyridopyrimidine ring. Conversely, in presence of a 4-F- or a 4-methoxy- benzyl in R2, the choice of a secondary amine seems to be preferred, as confirmed by the higher potency of **50**, **51** (R2 = ethylamine-containing chain; pEC_50_ = 6.70) and of **45** (pEC_50_ = 6.70), if compared to **42** (R2= piperazine-containing chain; pEC_50_ = 4.00) and **41** (pEC_50_ = 4.00), respectively.

**Figure 11:**
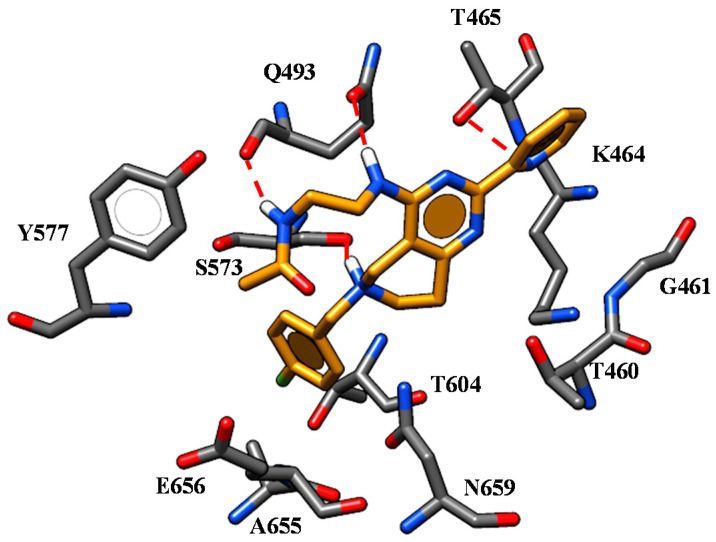
Docking mode of **50** (C atom; orange) within the CFTR *h*NBD1 domain. The most important residues are labelled.

Based on our docking calculations at the only NBD1 domain, the previously cited compounds **50**, **51** (pEC_50_ = 6.70) displayed a similar binding positioning (Figure 11). In particular, the pyridine nitrogen atom of these compounds was engaged in one H-bond with T465, while the protonated nitrogen atom of the tetrahydropyrido group formed polar contacts with S573.

Both the nitrogen atoms of the R2 substituent were H-bonded to Q493. Finally, the 4-F benzyl portion was projected towards F575, V603, T604 and A655 detecting hydrophobic interactions and π-π stackings. Within the 4-methoxy-benzyl derivatives, the most promising **45** (pEC_50_ = 6.70) displayed additional H-bonds involving T604 and S605 through its methoxy group, while the nitrogen atom of the pyrimidine core was engaged in polar contacts with T465 and K464. The imidazolyl group in R2 at the end of the aminoalkyl chain allowed compound **45** to exhibit further H-bonds with N659. The most relevant contacts detected for this series of correctors at the NBD1 domain are listed in Table S19.

**Figure 12:**
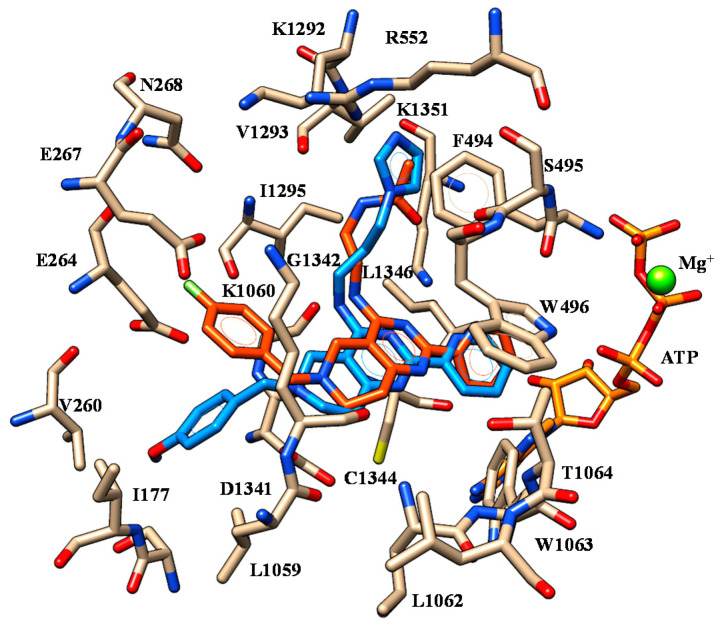
Selected docking mode of **45** (C atom; blue) and **50** (C atom; orange) within the modelled CFTR mutant. The most important residues are labelled.

Based on our F508del-CFTR model, docking calculations enlightened a common positioning for both **45** and **50**, as shown in Figure 12.

In particular, the terminal amide and imidazolyl group of **50** and **45** were engaged in one H-bond with V1293, resembling the same contacts previously mentioned for SUL-809 (see Figure 5). The nitrogen atom of the secondary amine was in any case H-bonded to G1342 while the protonated nitrogen atom of the tetrahydropyrido moiety was involved in a salt-bridge with D1341. While the R1 substituent of both the correctors was engaged in polar contacts with E264, E267 and S176, the pyridine groups exhibited one H-bond with K1351. Interestingly, these findings were in accordance with the SAR for tetrahydropyridopyrimidine in R2. Indeed, the introduction of a 2-pyridine substituent at the position 2, rather than a 3-pyridine or a 4-pyridine group, led to the most effective compounds. Accordingly, the tetrahydropyridopyrimidines **31**–**33** (R3 = 2-pyridine; pEC_50_ = 5.51–5.85) displayed higher potency than the related analogues **35**–**37** (R3 = 3-pyridine; pEC_50_ = 4.00) and also than **39**, **40** (R3 = 4-pyridine; pEC_50_ = 4.00). 

The role played by the introduction of rigid linker in R2, such as the piperidine or piperazine- containing groups featured by **41**, **43, 44, 46** (pEC_50_ = 4.00–6.40) was predicted by a different cluster of docking positioning when focusing only on the NBD1, if compared to the docking mode described for **45** and **50**. 

**Figure 13:**
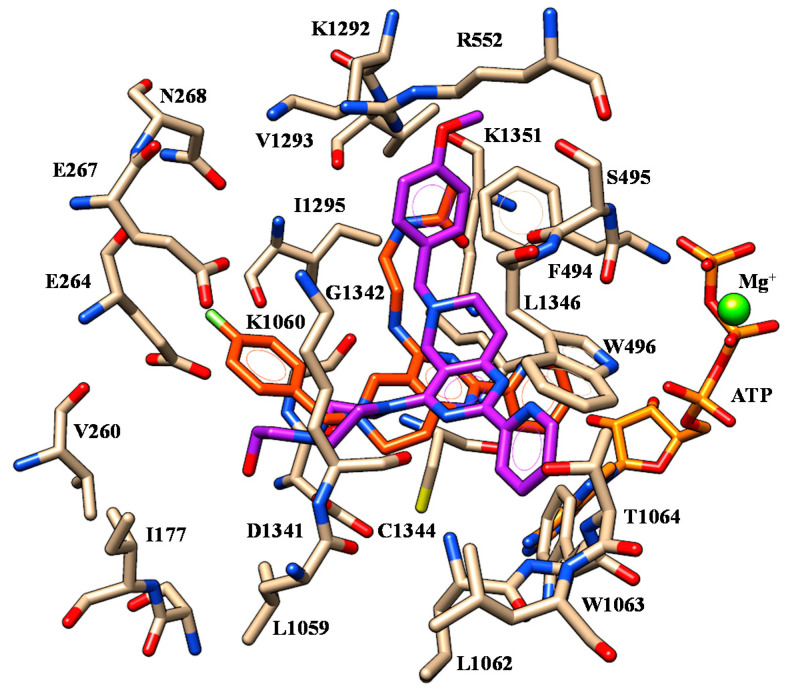
Selected docking mode of **43** (C atom; violet) and **50** (C atom; orange) within the modelled F508del-CFTR. The most important residues are labelled.

In regards to **43**, **46**, they detected interactions with G461 and K464 through their methoxy group and were established via further piperazine ring contacts with S573. In the case of compound **43** the hydroxyl group on the piperazine ring made an H-bond with N659, while compound **46** was H-bonded to the T604 residue. Finally, for both compounds the interactions of the piperidine moiety with E656 remained stable (S20). Taking into account the docking results obtained through the F508del-CFTR model, compound **43** was highly stabilized at the biological target thanks to salt-bridges involving the protonated piperazine and the hydroxyl moiety with D1341. The presence of the methoxy group at the R1 substituent guided the corrector near to V1293, featuring one H-bond, while the pyridine ring was engaged in π-π stacking with W496 and W1063 (Figure 13).

These findings support the highest potency of **45** and **50** bearing a flexible linker in R2, with respect to most of the analogues designed with a rigid spacer, such as **41**–**44** and **46** and **47,** with **45** and **50** endowed with the proper distance between the main core of the compound and the terminal H-bonding features, which allowed us to interact with G1342 and V1293.

Regarding cyanoquinoline (CQ) derivatives (**57**–**80**; pEC_50_ = 4.00–5.82), they included different compounds featuring the main bicyclic core tethered to a terminal (hetero)aryl-containing amide group by variable flexible or rigid spacers. In particular, CQ **57**–**68** (pEC_50_ = 4.00–5.82) and **69**–**74** (pEC_50_ = 4.00–5.52) exhibited the diaminoethyl- and the diaminopropyl- chains, while CQ **75**–**80** (pEC_50_ = 4.00) showed a piperazine linker (see S3 for the structures).

**Figure 14:**
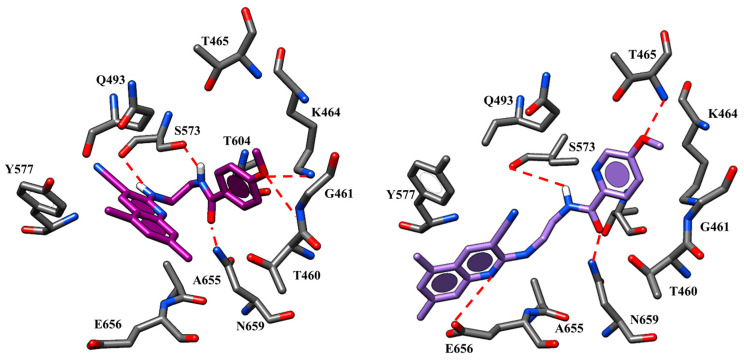
Docking mode of **59** (C atom; purple) and **67** (C atom; violet) within the CFTR *h*NBD1 domain. The most important residues are labelled.

Concerning the diaminoethyl-based derivatives, the presence of an H-bonding function at the *para* position of the terminal (hetero)aryl ring led to a lot of the most active compounds of this series (**59**, **64, 65,67**; pEC_50_ = 5.38–5.82). While **59**, **64** and **65** displayed a benzamide portion in R, CQ **67** was characterized by the picolinamide group (pEC_50_ = 5.57). According to our docking calculations within NBD1, these compounds maintained a comparable docking positioning, with the carboxamide of the linker engaged in H-bonds with S573 and N659 while the methoxy group on the terminal aromatic group moved in proximity to T465, G461 and K464, detecting H-bonds. The docking positioning related to compound **59** (pEC_50_ = 5.52) highlighted a number of H-bonds involving the N659-side chain as well as G461 and K464, through the methoxy group (Figure 14). The flexible spacer allowed the compound to be well-suited to fit the domain pocket, detecting hydrophobic contacts with the surrounding Y577 and A655.

CQ **67** occupied the channel crevice orienting the quinoline core towards Y577, A655 and G576, being H-bonded to the backbone of E656 residue (Figure 14). In this way, the compound displayed a similar positioning with that previously reported for the reference compound VX-809.

Indeed, the amide group was engaged in H-bonds with S573 and N659 while the methoxy group acted as a hydrogen acceptor function with respect to T465. The most relevant contacts detected for CQs at the NBD1 domain are listed in Table S21.

**Figure 15:**
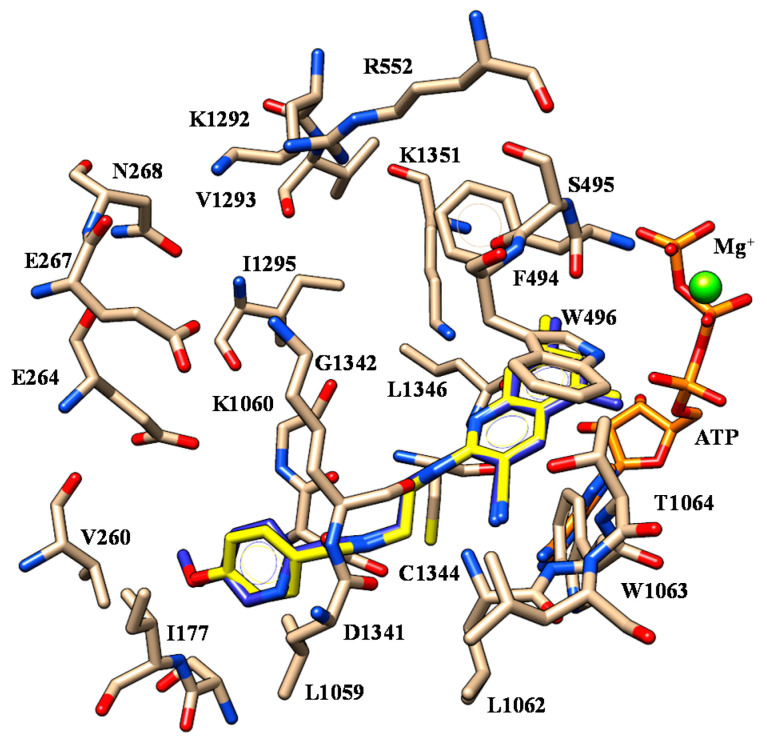
Selected docking mode of **59** (C atom; yellow) and **67** (C atom; blue) within the modelled F508del-CFTR. The most important residues are labelled.

At the whole protein, docking results underlined a common positioning of the two derivatives, with the -CN group H-bonded to L1062 and W1063, while the two nitrogen atoms of the spacer were engaged in H-bonds with K1060 and D1341 (see Figure 15).

This kind of positioning guarantees the proper hydrophobic contacts and π-π stacking with C1344 and W1063, F494, W496. Notably, the methoxy group of the R substituent was involved in contacts with I177.

These findings are in good agreement with the higher potency trend observed for the 4-subtituted **59** and **67** if compared to the unsubstituted **63** and **60** (pEC_50_ = 4.00), respectively. 

While moving the 4-methoxy substituent onto the *ortho* position of the (hetero)aryl group led to the modest corrector **58** (pEC_50_ = 4.96), the 3,4-dimethoxy benzamide-based analogue **65** (pEC_50_ = 5.82) proved to be the most potent within this series.

Derivative **65** maintained comparable positioning and key contacts with those described for the analogues **59** and **67** based on both the two docking sites (S22).

The introduction of the 1,3-diaminopropyl tether led the methoxy-substituted benzamide-based compounds **69–71** (pEC_50_ = 4.00–5.37) and the pyridine-containing **72–74** (pEC_50_ = 4.00–5.52), which proved to be less potent than the previously mentioned analogues bearing the diaminoethyl chain. Indeed, **69**–**71** and **72**-**74** featured lower pEC_50_ values if compared to **57–59** (pEC_50_ = 4.96–5.66) and to **61–63** (pEC_50_ = 4.00–5.57).

The most promising **73** (pEC_50_ = 5.52) highly mimicked the docking mode of **65** with guaranteed key contacts between the -CN and the spacer nitrogen atoms with L1062, W1063 and with K1060, D1341, respectively. In addition, the nicotinamide portion as terminal substituent was H-bonded to I177 (S22).

### 2.2. QSAR Analyses

In our previous work we reported QSAR analyses on a dataset of sixty-three F508del-CFTR correctors featuring cyanoquinolines, tetrahydropyridopyrimidines and thiazole scaffolds. This allowed us to identify eight descriptors explaining the corrector ability range of the collected library, with five and three of them 2D and 3D descriptors, respectively. These included parameters related to atoms and bound counts (b_single, a_IC and a_nH) and surface area descriptors (Vsurf_DD12 and Vsurf_W8). The connectivity-based descriptor chi1, the so-called distance matrix parameter weinerPol and the potential energy descriptor E_nb were also retained [30].

As we discussed, these promoted the design of branched scaffolds enriched with flexible substituents and quite polar moieties, within overall limited and bulky conformations. Based on the prediction performed with the model, we designed and synthesized a number of hybrids endowed with a promising CFTR rescue ability [30].

Herein we deemed interesting to refine our previous QSAR model including the chemical structure and the related corrector profile of the in-house hybrid correctors in the newly developed dataset. The derived optimized QSAR model, developed taking into account the compound positioning observed by docking calculations, is expected to deepen our knowledge on the SAR of this potent series of CFTR modulators.

In order to develop QSAR analyses, three hundred molecular descriptors (including 2D- and 3D- parameters) were calculated, by means of MOE software [41].

2D molecular descriptors are divided in seven subsets, representing physical properties (2D-I), subdivided surface areas (2D-II), atom and bond counts (2D-III), connectivity-based descriptors (2D-IV), partial charges descriptors (2D-V), pharmacophore features descriptors (2D-VI) and the so-called Adjacency and Distance Matrix Descriptors (2D-VII). 3D-descriptors include five groups, such as potential energy descriptors (3D-I), MOPAC descriptors (3D-II), Surface Area (3D-III), Volume and Shape Descriptors (3D-IV) and Conformation-Dependent Charge Descriptors (3D-V).

In this work, we applied the same statistical approach previously discussed [30].

The employed dataset included eighty compounds (**1**–**80**; see S1–S3), with compound **1** (pEC_50_ = 5.59) the well-known corrector VX-809 and derivatives **2**–**29** the aforementioned hybrids. Compounds **30**–**80** refer to the tetrahydropyridopyrimidines and CQs deeply investigated in the literature as F508del-CFTR correctors [24,25].

Model A was derived choosing manually the training set and test set compounds, taking into account the overall biological activity trend and structural variations.

Model B was obtained splitting the compounds into training and test sets using the Kennard-Stone design [42], probably the most popular algorithm for the selection of a subset of samples with a distribution as close as possible to the uniform distribution. In particular, the Kennard-Stone algorithm was performed adding the response vector (pEC_50_) as an additional column to the matrix of the descriptors in order to ensure that the training set compounds were distributed evenly within not only in the space defined by the descriptors, but also by the response values [43].

The refined predictive model A was calculated by dividing compounds **1**–**80** into a training set (**3**–**13**, **15**–**18**, **23**–**27**, **30**–**32**, **37**–**47**, **49**, **50**, **54**–**63**, **65**–**68**, **70**, **71**, **73**–**80**) to generate the QSAR model, and into a test set containing twenty derivatives (**1**, **2**, **14**, **19**–**22**, **28**, **29**, **33**–**36**, **48**, **51**–**53**, **64**, **69**, **72**), in order to evaluate the reliability of the mathematical relationship. In particular, the final model was generated by employing non-cross-validated PLS (partial least square) analysis to give a cross-validated r^2^ (r^2^_cv_) = 0.80, a non-cross-validated r^2^ (r^2^_ncv_) = 0.84, root mean square error (RMSE) = 0.333, a test set r^2^ (r^2^_pred_) = 0.78. The predicted and experimental rescue ability for all the derivatives are listed as tables together with the collected descriptors, as shown in the Supplementary Materials (S23–S24).

In regards to model B, twenty compounds (**4**, **6**, **7**, **8**, **16**, **17**, **24**, **25**, **33**, **35**–**37**, **40**, **58**, **60**, **63**, **70**, **77**, **78**, **80**) were assigned to the test set. It is possible to visualize the distribution of the compounds assigned to the training and test sets, in black and red respectively, in the plot shown in S25 which shows the PCA (principal component analysis) scores in the space of the first two principal components. The predicted and experimental rescue abilities for all the derivatives are listed as tables together with the collected descriptors, as shown in the Supplementary Materials (S26–S27).

In particular, the final model B was generated by employing non-cross-validated PLS analysis to give a cross-validated r^2^ (r^2^_cv_) = 0.78 a non-cross-validated r^2^ (r^2^_ncv_) = 0.86, root mean square error (RMSE) = 0.302, a test set r^2^ (r^2^_pred_) = 0.73.

A limited cluster of descriptors was selected using the QSAR-Contingency module implemented in MOE. Initially, the top fifty best-ranked parameters were retained based on the contingency scoring function, and then they were further filtered by re-iterative partial least square (PLS) analyses, including only those parameters showing the highest relative importance (RI) values.

Following this approach, we identified six descriptors related to the rescue ability profile of the collected library, with most of them 3D descriptors (see Table 1). Indeed, two descriptors fall in the 3D-I cluster, three in the 3D-V series and only one in the 2D-VI. Descriptor E_nb was the only one already retained in the previous published QSAR model.

All the 3D selected descriptors explain the polarity profile of the correctors, extended along the molecular surface, taking into account the conformational disposition of the ligands. On the other hand, these features also show specific and adequate requirements supporting the hydrophilic/hydrophobic balance onto the overall surface extent, as confirmed by the vsa_pol descriptor. This information is suggested for a proper overall dimension and shape of the ligand, turning in preferred branched and flexible substituents, over rigid, planar and much more extended groups. This is in harmony with the overall need for a prominent polarity profile, to be unraveled along a branched chemical structure. 

Quantitatively, the corrector ability of the compounds here studied is explained by the following equation Equations (1) and (2) by means of model A and B, respectively:

pEC_50_ = 12.91882−0.10591 × E_nb −0.01931 × ASA − 0.04699 × vsa_pol + 0.00260 × CASA^+^ + 0.00658 × CASA^−^ − 0.02034 × E_ang (1)

pEC_50_ = 12.40705 − 3.70703 × E_nb − 1.08439 × ASA − 0.51885 × vsa_pol + 2.77245 × CASA^+^ + 0.93650 × CASA^−^ − 0.152808 E_ang (2)

With the perspective of the results and taking into account the RI values related to the two models (A and B) the two QSAR approaches proved to be quite comparable.

Thus, a more detailed description of model A, featuring a higher predictive ability with respect to the test set is shown as follows.

**Figure 16:**
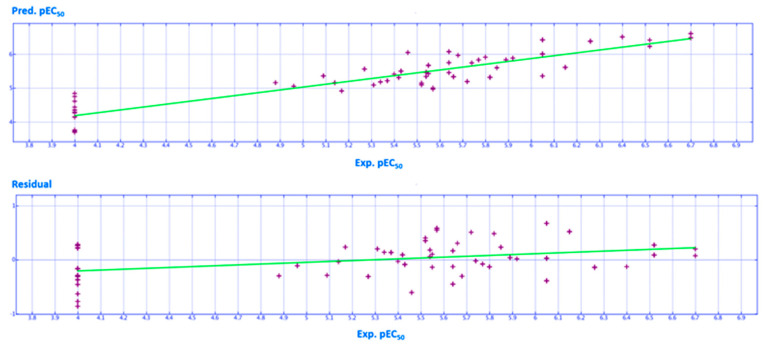
Distribution of the predicted (Pred.pEC_50_) and experimental (Exp.pEC_50_) potency values of the training set compounds (by violet crosses). Residual values are also reported, calculated as the difference between the Exp. pEC_50_ and the Pred. pEC_50_.

**Figure 17:**
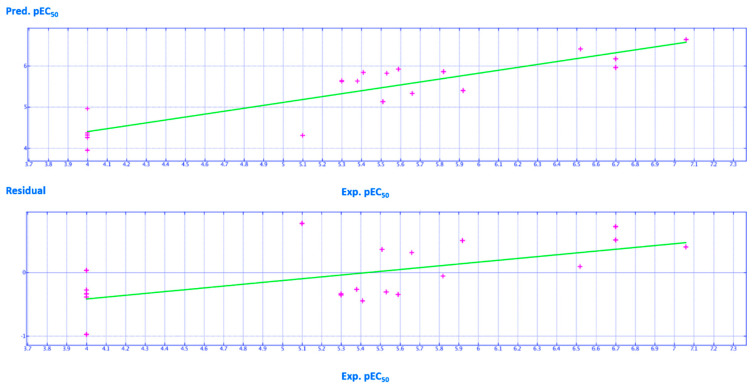
Distribution of the predicted (Pred.pEC_50_) and experimental (Exp.pEC_50_) potency values of the test set compounds (by magenta crosses). Residual values are also reported, calculated as the difference between the Exp.pEC_50_ and the Pred.pEC_50_.

A schematic representation of the high performance of the model is shown in Figure 16 and Figure 17, giving a perspective of the high performance of this model when predicting modest to promising F508del-CFTR correctors (4.50 < pEC_50_ <6.00), with the corresponding residual values near to zero.

**Figure 18:**
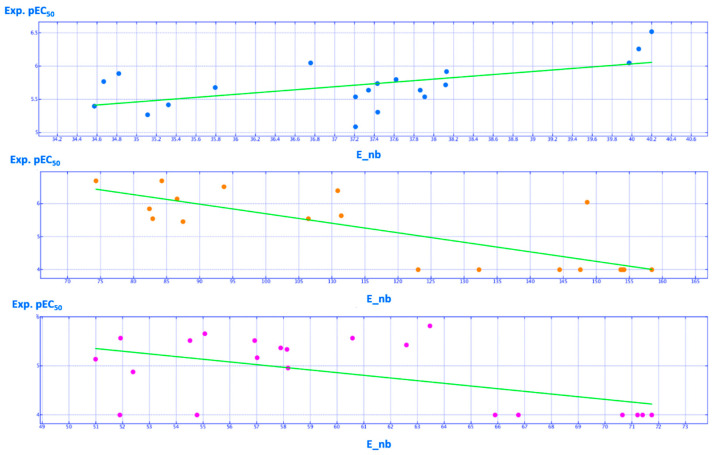
Distribution of the experimental (Exp.pEC_50_) potency values of the training set compounds based on the E_nb descriptor (Kcal/mol). Hybrid derivatives, tetrahydropyridopyrimidines and cyanoquinolines are reported as cyan, orange and magenta spheres, respectively.

Based on these data, E_nb and CASA^+^ represent the most relevant molecular descriptors affecting the biological activity trend of the VX-809 hybrids, of the tetrahydropyridopyrimidines and of the cyanoquinolines (see Table 1), featuring RI values > 0.6. As shown in Figure 18, the E_nb mean value within the hybrid series changes from 35 to 40 (Kcal/mol) for the modest and more promising correctors.

Increasing E_nb arises with the introduction of a polar electron-donor group tethered to the *meta* position of the phenyl ring rather than a bulky and hydrophobic group at the same position. Indeed, the 5-methyl thiazole **9** (pEC_50_ = 5.80; E_nb = 37.62 Kcal/mol) featured a lower E_nb value than the congener *m*-OH-phenyl substituted **18** (pEC_50_ = 5.92; E_nb = 38.13 Kcal/mol). In addition, **18** experienced higher E_nb than the *m*-Cl-phenyl substituted **8** (pEC_50_ = 5.64; E_nb = 37.86 Kcal/mol). Interestingly, the ideal value for this descriptor (E_nb = 40 Kcal/mol) was displayed by those hybrids exhibiting the ester moiety at the position 5 of the AAT. This was in agreement with the promising corrector ability of **5** (pEC_50_ = 6.05; E_nb = 39.97 Kcal/mol), **3** (pEC_50_ = 6.52; E_nb = 40.20 Kcal/mol) and **4** (pEC_50_ = 6.26; E_nb = 40.07 Kcal/mol) if compared to the unsubstituted analogues **11** (pEC_50_ = 5.54; E_nb = 37.21 Kcal/mol), **12** (pEC_50_ = 5.31; E_nb = 37.43 Kcal/mol) and **13** (pEC_50_ = 5.09; E_nb = 37.21 Kcal/mol), respectively.

Conceivably, it is thought that deep variations of E_nb value happen in the presence of branched or flexible substituents linked to the central scaffold of the corrector, leading to effective intra-molecular contacts, such as intra-hydrogen bonds and hydrophobic interactions. This kind of conformation is often experienced within the tetrahydropyridopyrimidines, featuring the most potent E_nb values spanning from 70 to 100 Kcal/mol, while the less effective analogues experience E_nb higher than 100 Kcal/mol (Figure 18). In particular, tetrahydropyridopyrimidines bearing flexible chains in R2 together with H-bond acceptor functions (such as **45** and **50**; pEC_50_ = 6.70) could be involved in intramolecular H bonds with the protonated nitrogen atom of the tetrahydropyridine ring, displaying proper levels in E_nb (**45**, E_nb = 84.29 Kcal/mol; **50**, E_nb = 74.34 Kcal/mol). 

On the contrary, more rigid tetrahydropyridopyrimidines bearing a piperazine ring tethered to the main bicyclic core often led to less promising derivatives exhibiting too high levels in the E_nb descriptor, over 100 Kcal/mol. This information is supported by the poor potency values of the piperazine-containing **38** (pEC_50_ = 4.00; E_nb = 153.96 Kcal/mol), **40**–**42** (pEC_50_ = 4.00; E_nb = 153.73–158.42 Kcal/mol), and **55**–**56** (pEC_50_ = 4.00; E_nb = 123.08–132.29 Kcal/mol). 

The most interesting CQs display an E_nb value between 50 and 65 Kcal/mol, while the less potent congeners show higher values of E_nb. In agreement with these data, CQs featuring basic and flexible ethylene diamine spacer, such as **65**–**68** (pEC_50_ = 5.17–5.82; E_nb = 57.02–63.47 Kcal/mol), featured lower E_nb than the modestly active analogues bearing a propylene diamine spacer in R1, such as **73**–**74** (pEC_50_ = 4.88–5.52; E_nb = 52.39–54.52 Kcal/mol) and the poorly active piperazine-containing **75**–**80** pEC_50_ = 4.00; E_nb = 65.90–71.22 Kcal/mol).

**Figure 19:**
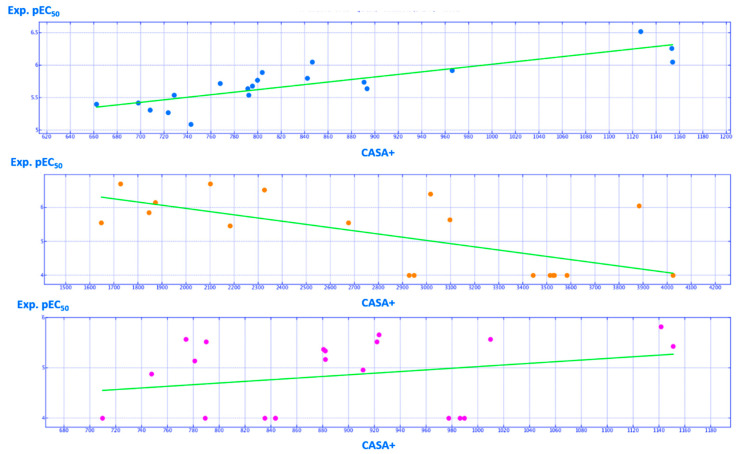
Distribution of the experimental (Exp.pEC_50_) potency values of the training set compounds based on the CASA^+^ descriptor (Å^2^). Hybrid derivatives, tetrahydropyridopyrimidines and cyanoquinolines are reported as cyan, orange and magenta spheres, respectively.

Concerning CASA^+^ (Figure 19), the overall electropositive profile experienced by hybrids as well as by CQs was quite comparable, revealing a mean value for the most promising derivatives around 1100 Å^2^.

In detail, the introduction of an electro-donor moiety such as the ester function at the position 5 of the thiazole increases the CASA+ descriptor values, with improvement of the corrector ability of the compounds. Accordingly, **5** (pEC_50_ = 6.05; CASA^+^ = 1154.36 Å^2^), **3** (pEC_50_ = 6.52; CASA^+^ = 1154.36 Å^2^) and **4** (pEC_50_ = 6.26; CASA^+^ = 1153.63 Å^2^) are endowed with higher potency values than the analogues unsubstituted at the same position of the five-membered ring **11** (pEC_50_ = 5.54; CASA^+^ = 728.87 Å^2^), **12** (pEC_50_ = 5.31; CASA^+^ = 708.34 Å^2^) and **13** (pEC_50_ = 5.09; CASA^+^ = 743.19 Å^2^). These findings support the high effectiveness of those hybrids bearing the hydroxyl group tethered to the phenyl ring at the position 4 of the thiazole if compared with the chlorine substituted analogues (compare **16** (pEC_50_ = 5.74; CASA^+^ = 890.95 Å^2^), **17** (pEC_50_ = 5.64; CASA^+^ = 893.51 Å^2^), **25** (pEC_50_ = 5.89; CASA^+^ = 804.04 Å^2^) and **18** (pEC_50_ = 5.92; CASA^+^ = 966.20 Å^2^) with **11** (pEC_50_ = 5.54; CASA^+^ = 728.87 Å^2^), **13** (pEC_50_ = 5.09; CASA^+^ = 743.19 Å^2^), **27** (pEC_50_ = 5.40; CASA^+^ = 662.58 Å^2^) and **8** (pEC_50_ = 5.64; CASA^+^ = 791.71 Å^2^)).

Taking into account the CQ series, the presence of methoxy- or dimethoxy- substitution onto the terminal aromatic ring allowed the increase of CASA^+^ descriptor accompanied by an overall improvement in terms of corrector ability. Similarly, the choice of benzamide moiety yield more promising compounds than the picolinamide or nicotinamide. The reliability of this information is confirmed by the higher pEC_50_ and by the CASA^+^ descriptor profile when the methoxy-substituted **57**–**59** (pEC_50_ = 4.96–5.66; CASA^+^ = 911.40-923.78 Å^2^) are compared to the unsubstituted heteroaryl-based analogues **60**–**62** (pEC_50_ = 4.00–5.57; CASA^+^ = 774.53–789.39 Å^2^) and to the benzamide analogue **63** (pEC_50_ = 4.00; CASA^+^ = 709.99 Å^2^). These data are shown and are maintained within the aforementioned series of cyanoquinolines, bearing the dimethyl spacer, as well as for the diaminopropyl- and the piperazine-containing analogues. In fact, the methoxy-substituted **71** (pEC_50_ = 5.37; CASA^+^ = 880.84 Å^2^) was more potent than **74** (pEC_50_ = 4.88; CASA^+^ = 747.92 Å^2^).

A number of the most promising tetrahydropyridopyrimidines were characterized by a value of CASA^+^ descriptor spanning from 1600 to 2300 Å^2^ (Figure 19), featuring the majority of the less potent (higher) values of this parameter. Conceivably, this information can be explained based on the prominent polarity of the main scaffold of this corrector series, being particularly enriched of nitrogen atoms and basic features.

Among them, the tetrahydropyridopyrimidines **50**, **51** (pEC_50_ = 6.70; CASA^+^ = 1729.20 Å^2^) display adequate CASA^+^ values thanks to their flexible basic chain in R2 as well as the methoxyphenyl-based analogue **45** (pEC_50_ = 6.70; CASA^+^ = 2102.26 Å^2^), also endowed with basic features. The distribution of the experimental potency values for the test set compounds with respect to the E_nb and CASA^+^ descriptors are shown in S28.

## 3. Discussion

In this work, two series of molecular docking calculations were performed concerning the different chemotypes of F508del-CFTR correctors herein investigated. Conceivably, based on the mean value of the docking scores (Score_mean_) obtained by the two approaches, VX-809 and related analogues (**1**–**29**; NBD1 Score_mean_ = −21.04; modelled CFTR Score_mean_ **=** −23.54) tetrahydropyridopyrimidines (**30**–**56**; NBD1 Score_mean_ = −17.60; modelled CFTR Score_mean_ = −26.67) and CQs (**57**–**80**; NBD1 Score_mean_ = −18.64; modelled CFTR Score_mean_ = −25.56) were predicted to bind at the NBD1:ICL4 interface. These findings suggest docking calculations at the modelled F508del-CFTR to be a more informative way to explore the putative mechanism of action of CFTR correctors, if compared to visual inspection focused on the only NBD1 domain.

In particular, docking results revealed a common positioning featured by the F508del-CFTR corrector VX-809 and by the highly related analogues ALK-809 and SUL-809 at the modelled CFTR. In regards to VX-809, the most applied and investigated drug for the treatment of cystic fibrosis, two salt-bridges involving R552, K1292 and H-bonding to K1060 resulted in support of the mechanism of action of this corrector, with VX-809 further stabilized at the NBD1:ICL4 interface by π-π stacking and van der Waals contacts with F494, W496 and W1063. Notably, some of us recently reported the docking mode of VX-809 by means of computational and experimental studies on the whole F508del-CFTR proteins, revealing polar and lipophilic contacts involving VX-809 and F494, W496 of NBD1 and W1060 and W1063 of ICL4 [18].

Deepening calculations performed onto the analogues ALK-809 and SUL-809 showed one H-bond with K1351 and further H-bonds to L1062, W1063 and K1060, L1062, W1064, respectively. This kind of positioning allowed arranging the hydrophobic terminal chains of the two correctors in the hydrophobic cavity delimited by W496, L1059, L1063, C1344 and L1346.

Along with this, comparable results were obtained for hybrids **2**–**29** (pEC_50_ = 5.08–7.06) as well as for other chemotypes endowed with CFTR corrector ability, such as tetrahydropyridopyrimidines **30**–**56** (pEC_50_ = 4.00–6.70) and cyanoquinolines **57**–**80** (pEC_50_ = 4.00–5.82). 

Tetrahydropyridopyrimidines were able to detect most of these contacts shared by SUL-809, and were also characterized by additional contacts between the protonated nitrogen atom of the tetrahydropyrido moiety and D1341 while the hydrophobic contacts with W496 and W1063 were maintained. Regarding CQ correctors, the most promising of them experienced the aforementioned key interactions previously reported for VX-809 and the related analogues, with the -CN group H-bonded to L1062 and W1063, while the two nitrogen atoms of the spacer were engaged in H-bonds with K1060 and D1341. More details about the most important contacts exhibited by all the corrector chemo-types herein studied are summarized in Table 2, Table 3 and Table 4.

The information obtained by QSAR analyses were in good agreement with those of docking results, revealing a consistent number of descriptors explaining the polarity profile of the correctors. Indeed, E_nb and CASA^+^ have been previously discussed as the most relevant parameters affecting the three series of CFTR modulators. Beyond these descriptors, the potency trend of hybrids was positively influenced by increasing values of E_ang, in accordance with the overall promising potency of the ester-containing analogues, as well as by a balanced electro-negative profile (S29).

These findings were demonstrated by the limited range of tolerated CASA^−^ and ASA^−^ parameters, experienced by the most potent hybrids (see S29). On the other hand, the pEC_50_ values of tetrahydropyridopyrimidines increased with these two descriptors, probably because of the high polarity of the main core of this corrector series featuring several nitrogen atoms and basic groups. As a result, the vsa_pol descriptor was also positively related to the corrector ability of this chemical series, supporting the need for a proper corrector shape endowed with a quite flexible and polar molecular surface to achieve corrector behavior (S30).

Accordingly, the most potent CQs were accompanied by increasing values of the vsa_pol parameter.

## 4. Materials and Methods

### 4.1. Molecular Docking

All the compounds **1**–**80** were manually built by the “MOE Builder” program and then were parameterized (AM1 partial charges as calculation method) and energy minimized by the “Energy Minimize Program” using MMFF94x forcefield and RMS (= root mean square) equal to 0.0001 Kcal/mol/A^2^ of the MOE compute module, to produce a single low-energy conformation for each ligand.

Molecular docking of all the investigated compounds was carried out on the crystallographic structures of the NBD1 domain of the F508del-CFTR (pdb code: 4WZ6) [38], which was prepared through the “Structure Preparation” program of the Molecular Operating Environment [41], and on the in-house model of F508del-CFTR protein. This model was managed based on superimposition of the whole protein (pdb code: 6MSM) [37] obtained by cryoelectron microscopy, with that of the mutated NBD1 domain (pdb code: 4WZ6) [38]. The modelled complete structure has been refined and minimized within MOE under the AMBER99 force field.

Molecular docking studies on VX-809 and related analogues were performed considering the putative binding site as proposed by the site finder module of MOE software.

The purpose of Site Finder is to calculate possible active sites in a receptor from the 3D atomic coordinates of the receptor. MOE’s Site Finder falls into the category of geometric methods, taking into account the relative positions and accessibility of the receptor atoms based on a rough classification of chemical type. The Site Finder methodology works on the Alpha Shapes represented by convex hulls as developed and described in the literature [44]. Then the module of this software identifies regions of tight atomic packing in order to filter out sites that are “too exposed” to solvent. This approach allows removing protrusions which are unlikely to be good active sites.

The derived preliminary sites are then classified based on their hydrophobic or hydrophilic profile. This coarse classification of chemical type is used to separate water sites from the more likely hydrophobic sites. Then, generation of alpha spheres on these sites by collecting sets of points using a modified Delaunay triangulation method allows deriving different spheres exhibiting different radii.

The collection of alpha spheres is pruned by eliminating those that correspond to inaccessible regions of the receptor as well as those that are too exposed to solvent. In addition, only the small alpha spheres are retained since these correspond to locations of tight atomic packing in the receptor.

Successively, alpha spheres are classified as either “hydrophobic” or “hydrophilic” depending on whether the sphere is in a good hydrogen bonding spot in the receptor.

Clustering the alpha spheres using a double-linkage clustering algorithm leads to a collection of sites where each site consists of one or more alpha spheres, at least one of which is hydrophobic.

All the collected sites are ranked according to their Propensity for Ligand Binding (PLB) score, which is based on the amino acid composition of the pocket as described in the literature [45].

Herein we considered the best ranked site by Site Finder for the following molecular docking calculations. Then, docking calculations were performed by means of LeadIT 2.1.8 software suite (www.biosolveit.com) starting from the *h*NBD1-VX-809 complex previously derived from automatic docking with MOE software. The docking algorithm is a state-of-the-art fragment docker: Ligands are split into so-called fragments, and an initial fragment (or combinations thereof) are placed into multiple places in the pocket and scored using a simple yet very fast pre-scoring scheme. From the n solutions placed, the ligand is further built up, fragment by fragment, and the interim solutions are scored against each other. The best scored survive the process, and those are delivered to the user. The initial idea relates back to the FlexX algorithm; however, many improvements have been made over the years, such as the “Single Interaction Scan” (SIS) placement that also finds solutions when there are only very few polar groups in a compound. The SIS algorithm uses virtual lines between protein and ligand interaction spots, and is trimmed for a very speedy rotation around these lines, thus generating solutions quickly for ligands of more hydrophobic character. The final docking poses were prioritized by the score values of the lowest energy pose of the compounds docked to the protein structure as shown in S4–S7. All ligands were refined and rescored by assessment with the algorithm HYDE, included in the LeadIT 2.1.8 software. The HYDE module considers dehydration enthalpy and hydrogen bonding [46,47]. In summary, HYDE was conceived to reflect the experimentally observed binding contributions due to hydrogen bonding and the hydrophobic effect in a very accurate way. HYDE works on new terms for the dehydration of idealized polar and apolar functional groups and provides a simple explanation for the main characteristics of inter-molecular interaction in aqueous solution, including the hydrophobic effect, dehydration penalties, and hydrogen bonding. Because the hydrophobic effects are quantitatively described as the free dehydration enthalpy of an apolar function, the noncovalent contributions to intermolecular interactions in aqueous solutions and ∆G_binding_ can be calculated by considering only two main terms: a) the dehydration of the interacting molecular interfaces, ∆G_dehydration_, and b) the vacuum hydrogen bond energies between interacting hydrogen bond functions. The current version of HYDE works best for accurately placed ligands in which the ligand pose has been generated using the geometric parameters derived from small-molecule crystallographic data.

### 4.2. QSAR Analysis

The corrector activity of the compounds was evaluated by means of fluorescence assay for CFTR activity. This protocol allowed us to obtain dose-response relationships from each experiment [24,25,30]. On this basis, the correctors have been selected for the dataset and the related pEC_50_ values have been calculated. QSAR studies were performed based on calculations of three hundred molecular descriptors, including 2D and 3D parameters, by means of MOE software. 2D molecular descriptors are defined to be numerical properties that can be calculated from the connection table representation of a molecule (e.g., elements, formal charges and bonds, but not atomic coordinates). 2D descriptors are, therefore, not dependent on the conformation of a molecule and are most suitable for large database studies. They include descriptors related to physical properties, subdivided surface areas, atom and bond counts, connectivity-based descriptors, partial charges descriptors, pharmacophore feature descriptors and the so-called Adjacency and Distance Matrix Descriptors. The 3D-descriptors consist of potential energy descriptors, MOPAC descriptors, Surface Area, Volume and Shape Descriptors and Conformation Dependent Charge Descriptors.

Afterwards, 302 molecular descriptors (2D and 3D) were computed by MOE and the resulting matrix was submitted to the statistical analyses and quantitative structure activity relationships (QSAR), objects of the present work. In addition, QuaSAR-Contingency was employed for pruning molecular descriptors, while the QSAR analysis module of MOE software was applied to generate the final mathematical models [48,49,50].

In order to perform quantitative structure activity relationship (QSAR) studies, sixty correctors were included into the training set for model generation, and the other ones into the test set for model validation. In regards to model A, the compounds were divided into the training and the test set pools manually, based on representative criteria of the overall biological activity trend and structural variations. Model B was developed using the Kennard-Stone algorithm in order to split the compounds into two representative sets (i.e., training set and a test set) in terms of chemical structures and biological activity. Principal component analysis (PCA) [51] was used, as a multivariate display method, in order to visualize the data structure.

QSAR was performed by the application of various iterations of partial least-squares (PLS) multivariate analysis, considering the molecular descriptors as independent variables and corrector pEC_50_ values as dependent variables. At each iteration, the relative importance of every descriptor in influencing corrector ability was calculated, therefore the less important ones were discarded in the following PLS analysis until the generation of the final linear regression model. At each PLS, Leave One Out method was used to check the internal predictability of the derived models.

The predictive ability of the derived model was evaluated for the test set compounds (expressed as r^2^_pred_), by using the following Equation (3):

r^2^_pred_ = (SD−PRESS)/SD (3)

SD is the sum of the squared deviations between the biological activities of the test set molecules and the mean activity of the training set compounds and PRESS is the sum of the squared deviations between the observed and the predicted activities of the test set compounds.

## 5. Conclusions

Herein we reported deepened molecular docking studies comparing the results obtained within the experimental data on the human NBD1 domain of the F508del-CFTR with those derived by taking into account the in-house modelled whole F508del-CFTR. These studies were performed including the series of AAT-VX-809 hybrids as well as cyanoquinolines and tetrahydropyridopyrimidines reported in the literature, acting as correctors. The results pointed out mandatory interactions with key residues of NBD1 such as F494 and W496 as well as amino acids belonging to the ICL4 domain (K1060 and W1063). Further studies related to site specific mutagenesis represent an urgent need to proceed with optimized computational studies and to definitively validate these results.

Our results were supported by the refined QSAR model herein proposed focusing on the chemical descriptors CASA^+^ and E_nb as related to the corrector ability of these derivatives. 

These data are expected to pave the way for a further design process towards more potent and selective F508del CFTR correctors.

## Figures and Tables

**Table 1:** Selected descriptor and related relative importance (RI) values based on model A and *model B (in italic)*.

Descriptor	Type	Series	RI
E_nb	Value of the potential energy related to non-bonded terms	3D-I	1.000000 (*1.000000*)
CASA^+^	Positive charge weighted surface area, ASA+ times max {*q_i_* > 0} [a]	3D-V	0.663223 (*0.747889*)
ASA^−^	Water accessible surface area of all atoms with negative partial charge (strictly less than 0)	3D-V	0.305252 (*0.292524*)
E_ang	Angle bend potential energy	3D-I	0.232607 (*0.152808*)
CASA^−^	Negative charge weighted surface area, ASA- times max {*q_i_* < 0}	3D-V	0.229658 (*0.252628*)
vsa_pol	Approximation to the sum of VDW surface areas (Å^2^) of polar atoms (atoms that are both hydrogen bond donors and acceptors)	2D-VI	0.136024 (*0.139963*)

**Table 2:** Pattern of the most important interactions within the modelled F508del-CFTR protein observed for the most promising hybrids, chosen as representative of this series of correctors.

Corrector	H-Bonds		π-π Stacking	
	**Amino Acid Residues**	**Ligand Portion**	**Amino Acid Residues**	**Ligand Portion**
**2**	K1060	Oxygen atom of benzodioxole group	F494	Benzodioxole moiety
	S495	Nitrogen atom of the carboxamide moiety	W496 W1063	Phenyl ring
	W1063	Ester oxygen atom		
**3**	K1060	Oxygen atom of benzodioxole group	F494	Benzodioxole moiety
	S495	Nitrogen atom of the carboxamide moiety	W496 W1063	Phenyl ring
	W1063	Ester oxygen atom		
**4**	K1060	Oxygen atom of benzodioxole group	F494	Benzodioxole moiety
	S495	Nitrogen atom of the carboxamide moiety	W496 W1063	Phenyl ring
	W1063	Ester oxygen atom		
**6**	I1295	Benzodioxole moiety	F494	Benzodioxole moiety
	K1351	Carboxamide oxygen atom	W496 W1063	Phenyl ring
**10**	I1295	Benzodioxole moiety	F494	Benzodioxole moiety
	K1351	Carboxamide oxygen atom	W496 W1063	Phenyl ring
**11**	I1295	Benzodioxole moiety	F494	Benzodioxole moiety
	K1351	Carboxamide oxygen atom	W496 W1063	Phenyl ring
**18**	I1295	Benzodioxole moiety	F494	Benzodioxole moiety
	K1351	Carboxamide oxygen atom	W496 W1063	Phenyl ring
	K1060	Methoxy group		

**Table 3:** Pattern of the most important interactions observed within the tetrahydropyridopyrimidines series at the modelled F508del-CFTR protein.

Corrector	H-Bonds		π-π Stacking	
	**Amino Acid Residues**	**Ligand Portion**	**Amino Acid Residues**	**Ligand Portion**
**43**	V1293	Methoxy group	W496 W1063	Pyridine ring
	D1341	Nitrogen atom of Tetrahydropyrido-moiety		
**45**	V1293	Terminal amide Imidazolyl group	W496 W1063	Pyridine ring
	D1341	Nitrogen atom of Tetrahydropyrido-moiety		
	G1342	Nitrogen atom of secondary amine		
	K1351	Pyridine group		
**46**	V1293	Methoxy group	W496 W1063	Pyridine ring
	D1341	Nitrogen atom of Tetrahydropyrido-moiety		
**50**	V1293	Terminal amide Imidazolyl group	W496 W1063	Pyridine ring
	G1342	Nitrogen atom of secondary amine		
	K1351	Pyridine group		
	D1341	Nitrogen atom of Tetrahydropyrido-moiety		
**51**	V1293	Terminal amide Imidazolyl goup	W496 W1063	Pyridine ring
	G1342	Nitrogen atom of secondary amine		
	K1351	Pyridine group		
	D1341	Nitrogen atom of Tetrahydropyrido-moiety		

**Table 4:** Pattern of the most important interactions within the modelled F508del-CFTR protein observed for the most promising CQs, chosen as representative of this series of correctors.

Corrector	H-Bonds		π-π Stacking	
	**Amino acid residues**	**Ligand Portion**	**Amino Acid Residues**	**Ligand Portion**
**59**	L1062 W1063	-CN group	W1063 F494 W496	Quinoline core
	K1060 D1341	Spacer nitrogen atoms		
**65**	L1062 W1063	-CN group	W1063 F494 W496	Quinoline core
	K1060 D1341	Spacer nitrogen atoms		
**67**	L1062 W1063	-CN group	W1063 F494 W496	Quinoline core
	K1060 D1341	Spacer nitrogen atoms		
**73**	L1062 W1063	-CN group	W1063 F494 W496	Quinoline core
	K1060 D1341	Spacer nitrogen atoms		
	I177	Nicotinamide portion		

## Supplementary Materials

Supplementary materials can be found at https://www.mdpi.com/1422-0067/21/21/8084/s1.

## Abbreviations

AAT	aminoarylthiazole
CF	cystic fibrosis
CFTR	cystic fibrosis transmembrane conductance regulator
CQ	cyanoquinoline
F508de	deletion of phenylalanine at position 508
NBD1	nucleotide binding domain 1
NBD2	nucleotide binding domain 2
PDB	Protein data bank
