# Supplementary material for: Molecular Docking and QSAR Studies as Computational Tools Exploring the Rescue Ability of F508del CFTR Correctors
Source: Int J Mol Sci. 2020 Oct 29;21(21):8084. doi: 10.3390/ijms21218084 (PMC7663332; doi:10.3390/ijms21218084)

International Journal of *Molecular Sciences*

MDPI

SUPPLEMENTARY MATERIALS

S1. Chemical structure and biological potency of the hybrids 2-29 as F508del CFTR correctors.

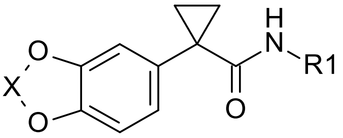

Cp.	X	R₁	pEC50
**2**	$-CH_2$	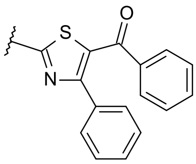	7.06
**3**	$-CH_2$	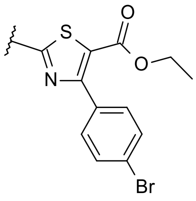	6.52
**4**	$-CH_2$	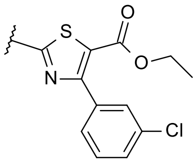	6.26
**5**	$-CH_2$	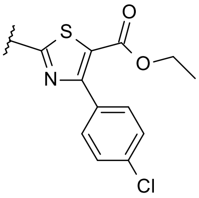	6.05
**6**	$-CH_2$	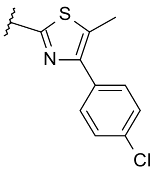	5.54
**7**	$-CH_2$	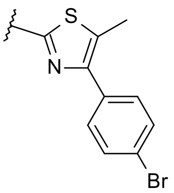	5.72

**8**	$-CH_2$	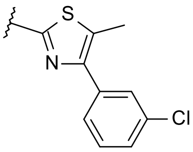	5.64
**9**	$-CH_2$	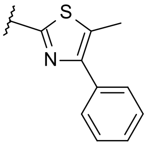	5.80
**10**	$-CH_2$	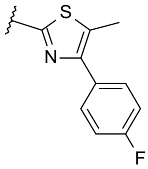	6.05
**11**	$-CH_2$	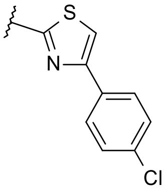	5.54
**12**	$-CH_2$	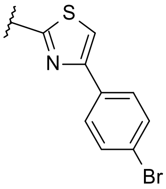	5.31
**13**	$-CH_2$	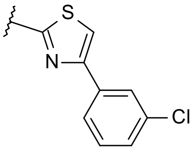	5.09
**14**	$-CH_2$	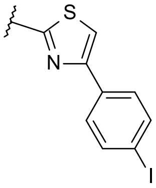	5.92
**15**	$-CH_2$	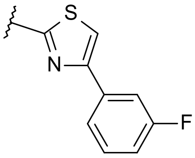	5.68
**16**	$-CH_2$	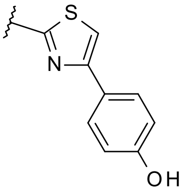	5.74

**17**	$-CH_2$	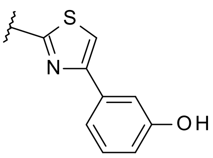	5.64
**18**	$-CH_2$	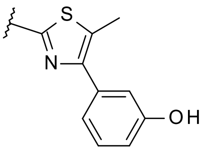	5.92
**19**	$-CH_2$	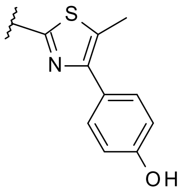	5.41
**20**	$-CH_2$	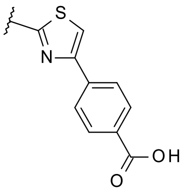	5.30
**21**	$-CH_2$	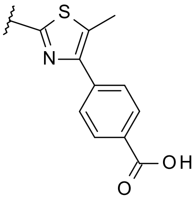	5.53
**22**	$-CH_2$	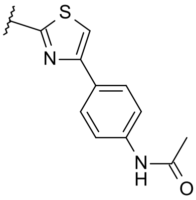	5.30
**23**	$-CF_2$	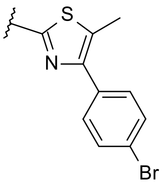	5.42
**24**	$-CF_2$	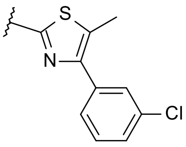	5.27
**25**	$-CF_2$	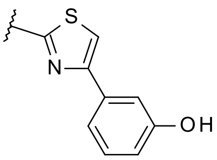	5.89

**26**	$-CF_2$	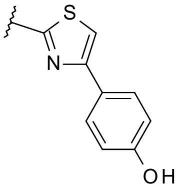	5.77
**27**	$-CF_2$	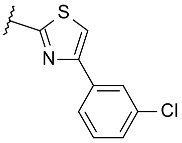	5.40
**28**	$-CH_2$	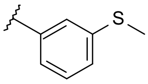	5.10
**29**	$-CH_2$	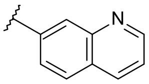	5.66

S2. Chemical structure and biological potency of the tetrahydropyridopyrimidines **30-56** as F508del CFTR correctors.

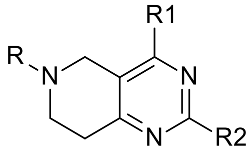

Cp.	R	R_1	R_2	pEC_{50}
**30**	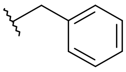	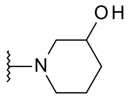	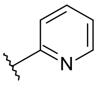	5.55
**31**	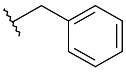	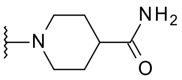	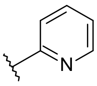	5.85
**32**	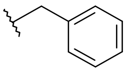	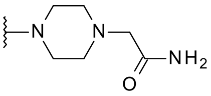	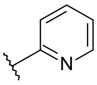	5.55
**33**	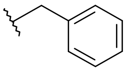	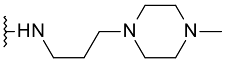	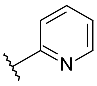	5.51
**34**	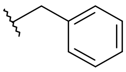	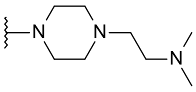	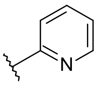	5.82

**35**	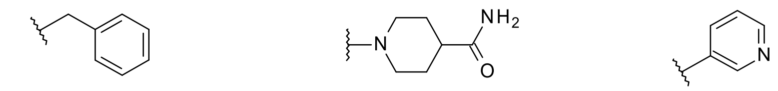	4.00
**36**	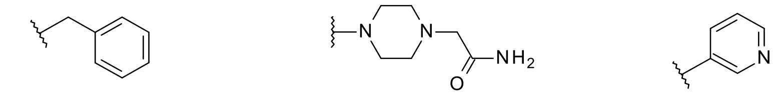	4.00
**37**	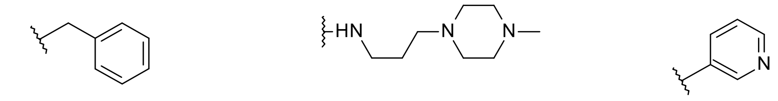	4.00
**38**	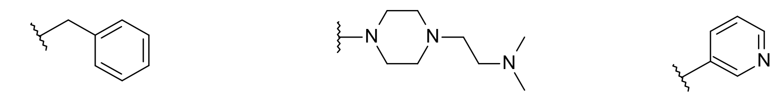	4.00
**39**	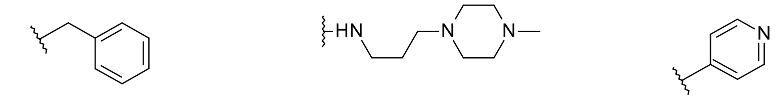	4.00
**40**	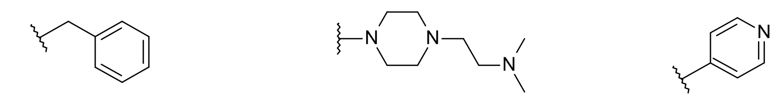	4.00
**41**	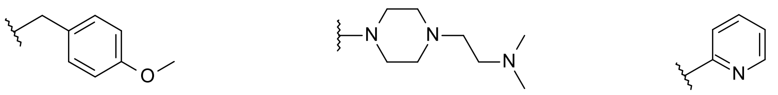	4.00
**42**	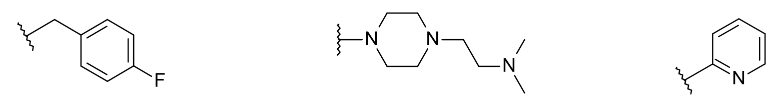	4.00
**43**	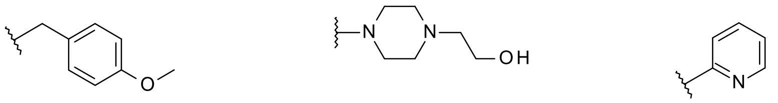	6.40
**44**	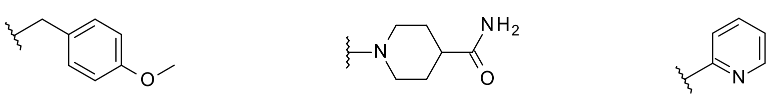	5.46
**45**	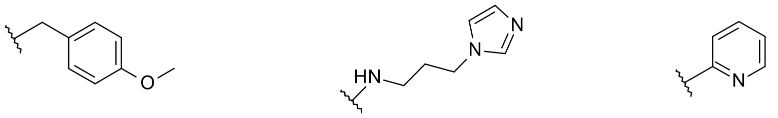	6.70
**46**	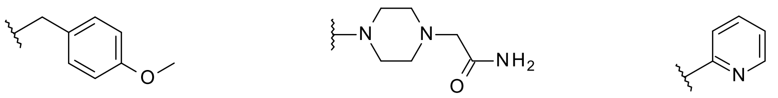	5.64
**47**	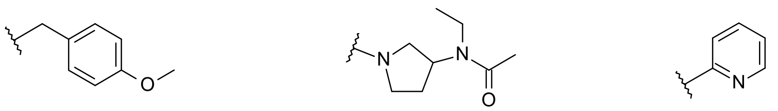	6.52
**48**	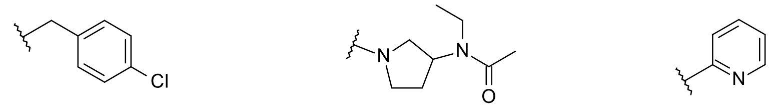	4.00

**49**	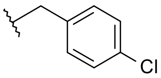	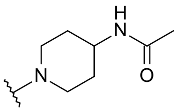	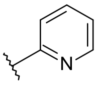	6.15
**50**	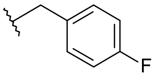	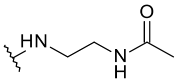	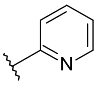	6.70
**51**	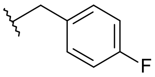	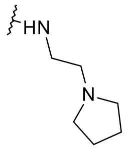	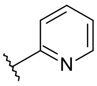	6.70
**52**	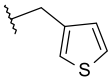	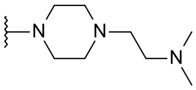	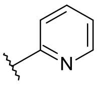	6.70
**53**	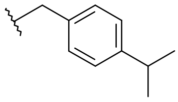	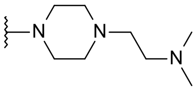	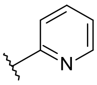	6.52
**54**	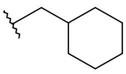	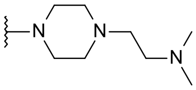	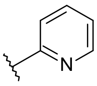	6.05
**55**	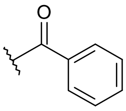	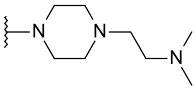	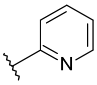	4.00
**56**	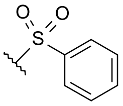	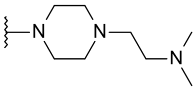	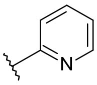	4.00

S3. Chemical structure and biological potency of cyanoquinolines **57-80** as F508del CFTR correctors.

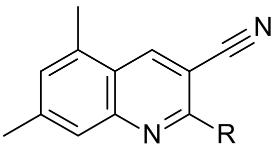

Cp.	R	pEC50
**57**	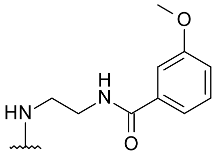	5.66

**58**	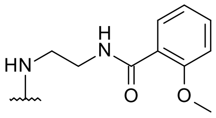	4.96
**59**	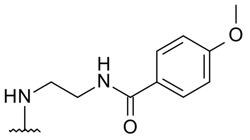	5.52
**60**	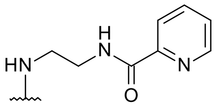	4.00
**61**	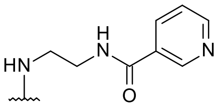	5.57
**62**	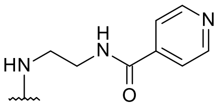	5.14
**63**	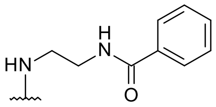	4.00
**64**	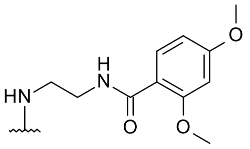	5.38
**65**	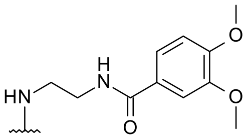	5.82
**66**	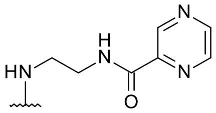	5.17
**67**	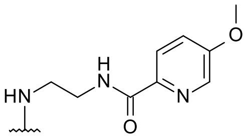	5.57
**68**	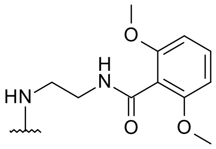	5.43
**69**	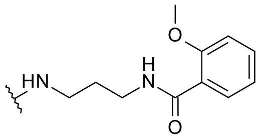	4.00

**70**	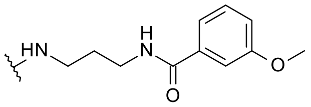	5.34
**71**	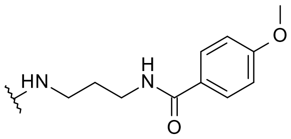	5.37
**72**	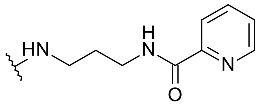	4.00
**73**	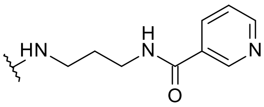	5.52
**74**	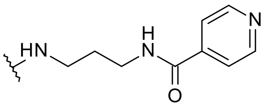	4.88
**75**	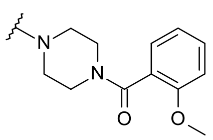	4.00
**76**	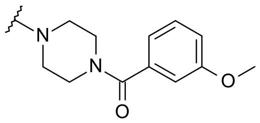	4.00
**77**	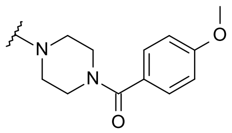	4.00
**78**	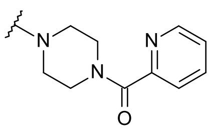	4.00
**79**	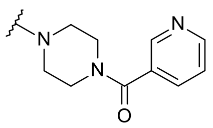	4.00
**80**	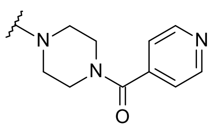	4.00

S4. (*left*) Superimposition of the modelled F508del-CFTR (*blu*) onto the 4WZ6 pdb code (*yellow*) is shown. (*right*)A detail of the comparison is depicted. Mg^{2+} and ATP are reported in spacefill mode while the docked VX-809 at the whole protein is shown in stick (C atom; green).

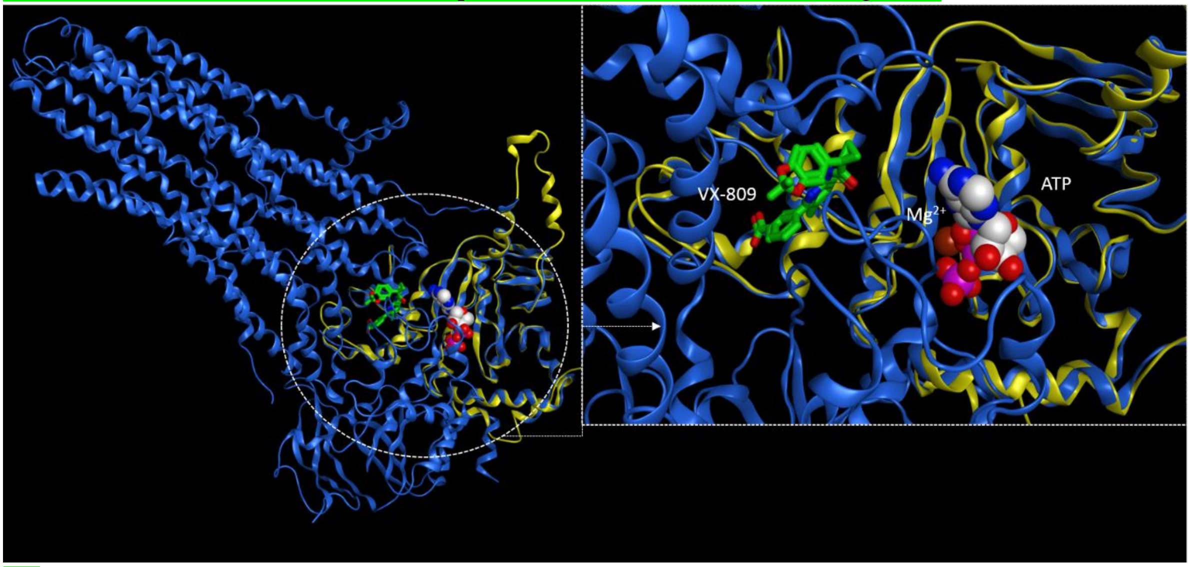

S5. Binding affinity values obtained by molecular docking studies of **VX-809**, **ALK-809**, **SUL-809.**

**hNBD1/(*CFTR*)-Corrector Complex (LeadIT)**	**Binding Affinity Energy ΔG (kJ/mol)**		**Score**	
hNBD1/(*CFTR*)-**VX-809**	-23.0	*-25.0*	-21.5663	*-35.6469*
hNBD1/(*CFTR*)-**ALK-809**	-21.0	*-22.0*	-21.9843	*-31.4063*
hNBD1/(*CFTR*)-**SUL-809**	-24.0	*-22.0*	-24.5672	*-35.2638*

S6. Binding affinity values obtained by molecular docking studies of hybrid correctors.

**hNBD1/(*CFTR*)-Corrector Complex (LeadIT)**	**Binding Affinity Energy ΔG (kJ/mol)**		**Score**	
hNBD1/(*CFTR*)-**2**	-19.0	*-21.0*	-24.8888	*-28.4416*
hNBD1/(*CFTR*)-**3**	-18.0	*-16.0*	-24.3256	*-23.3950*
hNBD1/(*CFTR*)-**4**	-18.0	*-20.0*	-23.4393	*-23.2050*
hNBD1/(*CFTR*)-**5**	-19.0	*-21.0*	-24.4022	*-23.3299*

hNBD1/(*CFTR*)-**6**	-14.0	*-12.0*	-21.1282	*-23.0777*
hNBD1/(*CFTR*)-**7**	-10.0	*-13.0*	-21.3208	*-23.0264*
hNBD1/(*CFTR*)-**8**	-15.0	*-17.0*	-19.6742	*-23.5280*
hNBD1/(*CFTR*)-**9**	-16.0	*-18.0*	-19.2015	*-22.9820*
hNBD1/(*CFTR*)-**10**	-14.0	*-12.0*	-21.3407	*-22.7400*
hNBD1/(*CFTR*)-**11**	-14.0	*-16.0*	-22.1238	*-22.0175*
hNBD1/(*CFTR*)-**12**	-15.0	*-17.0*	-20.4414	*-21.9677*
hNBD1/(*CFTR*)-**13**	-14.0	*-12.0*	-21.5660	*-22.6050*
hNBD1/(*CFTR*)-**14**	-17.0	*-19.0*	-21.9788	*-22.3278*
hNBD1/(*CFTR*)-**15**	-16.0	*-14.0*	-21.4102	*-22.9320*
hNBD1/(*CFTR*)-**16**	-17.0	*-19.0*	-20.5806	*-24.5206*
hNBD1/(*CFTR*)-**17**	-14.0	*-16.0*	-19.8188	*-25.1833*
hNBD1/(*CFTR*)-**18**	-15.0	*-13.0*	-20.4794	*-25.7335*
hNBD1/(*CFTR*)-**19**	-12.0	*-10.0*	-20.1269	*-25.5958*
hNBD1/(*CFTR*)-**20**	-13.0	*-15.0*	-20.3380	*-34.2077*
hNBD1/(*CFTR*)-**21**	-16.0	*-18.0*	-19.6181	*-33.4221*
hNBD1/(*CFTR*)-**22**	-14.0	*-16.0*	-18.7362	*-30.3549*
hNBD1/(*CFTR*)-**23**	-14.0	*-12.0*	-17.4095	*-22.7536*
hNBD1/(*CFTR*)-**24**	-16.0	*-18.0*	-16.1613	*-23.1016*
hNBD1/(*CFTR*)-**25**	-10.0	*-12.0*	-22.8956	*-25.9998*

hNBD1/(*CFTR*)-**26**	-17.0	*-19.0*	-20.3272	*-25.7090*
hNBD1/(*CFTR*)-**27**	-16.0	*-17.0*	-20.0106	*-22.7284*
hNBD1/(*CFTR*)-**28**	-17.0	*-18.0*	-21.8150	*-20.3860*
hNBD1/(*CFTR*)-**29**	-16.0	*-15.0*	-23.5860	*-22.0357*

S7. Binding affinity values obtained by molecular docking studies of tetrahydropyridopyrimidines**.**

S8. Binding affinity values obtained by molecular docking studies of cyanoquinolines.

**hNBD1/(*CFTR*)-Corrector Complex (LeadIT)**	**Binding Affinity Energy ΔG (kJ/mol)**		**Score**	
hNBD1/(*CFTR*)-**30**	-20.0	*-18.0*	-25.6464	*-27.9264*
hNBD1/(*CFTR*)-**31**	-18.0	*-16.0*	-21.2608	*-37.9208*
hNBD1/(*CFTR*)-**32**	-19.0	*-17.0*	-20.3588	*-32.6728*
hNBD1/(*CFTR*)-**33**	-18.0	*-16.0*	-22.3059	*-37.3057*
hNBD1/(*CFTR*)-**34**	-17.0	*-15.0*	-19.4212	*-38.2231*
hNBD1/(*CFTR*)-**35**	-12.0	*-11.0*	-12.8528	*-28.3428*
hNBD1/(*CFTR*)-**36**	-10.0	*-12.0*	-12.8948	*-29.4833*
hNBD1/(*CFTR*)-**37**	-11.0	*-13.0*	-11.7806	*-35.3428*
hNBD1/(*CFTR*)-**38**	-11.0	*-14.0*	-10.7854	*-29.9647*
hNBD1/(*CFTR*)-**39**	-12.0	*-13.0*	-10.3905	*-29.9688*
hNBD1/(*CFTR*)-**40**	-13.0	*-15.0*	-10.5560	*-28.7030*

**hNBD1/(*CFTR*)-Corrector Complex (LeadIT)**	**Binding Affinity Energy ΔG (kJ/mol)**		**Score**	
hNBD1/(*CFTR*)-**57**	-20.0	*-22.0*	-23.1906	*-31.3780*
hNBD1/(*CFTR*)-**58**	-11.0	*-13.0*	-14.2463	*-27.0360*
hNBD1/(*CFTR*)-**59**	-14.0	*-16.0*	-17.9541	*-24.8135*
hNBD1/(*CFTR*)-**60**	-20.0	*-18.0*	-20.2775	*-31.9695*
hNBD1/(*CFTR*)-**61**	-19.0	*-17.0*	-20.9974	*-27.0538*
hNBD1/(*CFTR*)-**62**	-16.0	*-18.0*	-18.8641	*-25.6758*
hNBD1/(*CFTR*)-**63**	-19.0	*-20.0*	-20.8618	*-26.0331*
hNBD1/(*CFTR*)-**64**	-20.0	*-18.0*	-20.1370	*-25.3409*
hNBD1/(*CFTR*)-**65**	-19.0	*-16.0*	-19.8917	*-25.2137*
hNBD1/(*CFTR*)-**66**	-20.0	*-18.0*	-21.4915	*-28.9083*

hNBD1/(*CFTR*)-[illegible]	[illegible]	[illegible]	[illegible]	[illegible]
hNBD1/(*CFTR*)-[illegible]	[illegible]	*-14.0*	[illegible]	[illegible]
hNBD1/(*CFTR*)-[illegible]	-18.0	[illegible]	[illegible]	[illegible]
hNBD1/(*CFTR*)-[illegible]	[illegible]	[illegible]	[illegible]	[illegible]
hNBD1/(*CFTR*)-[illegible]	-16.0	*-18.0*	[illegible]	[illegible]
hNBD1/(*CFTR*)-[illegible]	[illegible]	[illegible]	[illegible]	[illegible]
hNBD1/(*CFTR*)-[illegible]	-20.0	[illegible]	[illegible]	[illegible]
hNBD1/(*CFTR*)-[illegible]	[illegible]	[illegible]	[illegible]	[illegible]
hNBD1/(*CFTR*)-[illegible]	[illegible]	[illegible]	[illegible]	[illegible]
hNBD1/(*CFTR*)-[illegible]	[illegible]	[illegible]	[illegible]	[illegible]
hNBD1/(*CFTR*)-[illegible]	[illegible]	[illegible]	[illegible]	[illegible]
hNBD1/(*CFTR*)-[illegible]	[illegible]	[illegible]	[illegible]	[illegible]
hNBD1/(*CFTR*)-[illegible]	[illegible]	[illegible]	[illegible]	[illegible]
hNBD1/(*CFTR*)-[illegible]	[illegible]	[illegible]	[illegible]	[illegible]
hNBD1/(*CFTR*)-55	-9.0	-11.0	-10.1093	*-33.5767*
hNBD1/(*CFTR*)-56	-11.0	-13.0	-10.5794	*-29.5519*

S9. Distribution of the score value obtained by the molecular docking at the NBD1 domain, concerning the VX-809 analogues (magenta dots), tetrahydropyridopyrimidines (red dots) and cyanoquinolines (cyan dots) with respect to their experimental potency values (Exp.pEC_{50}).

.

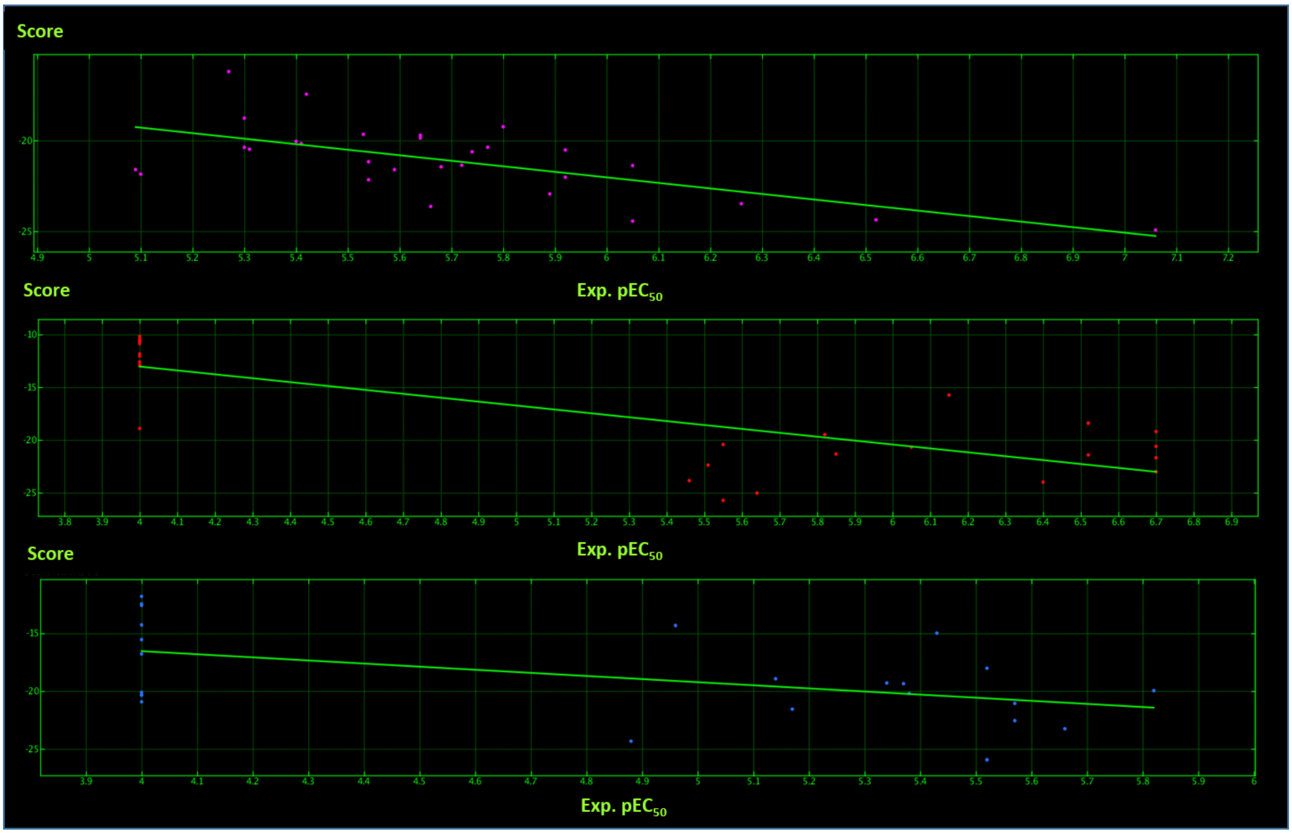

S10. Distribution of the score value obtained by the molecular docking at the modelled F508del-CFTR, concerning the VX-809 analogues (magenta dots), tetrahydropyridopyrimidines (red dots) and cyanoquinolines (cyan dots) with respect to their experimental potency values (Exp.pEC_{50}).

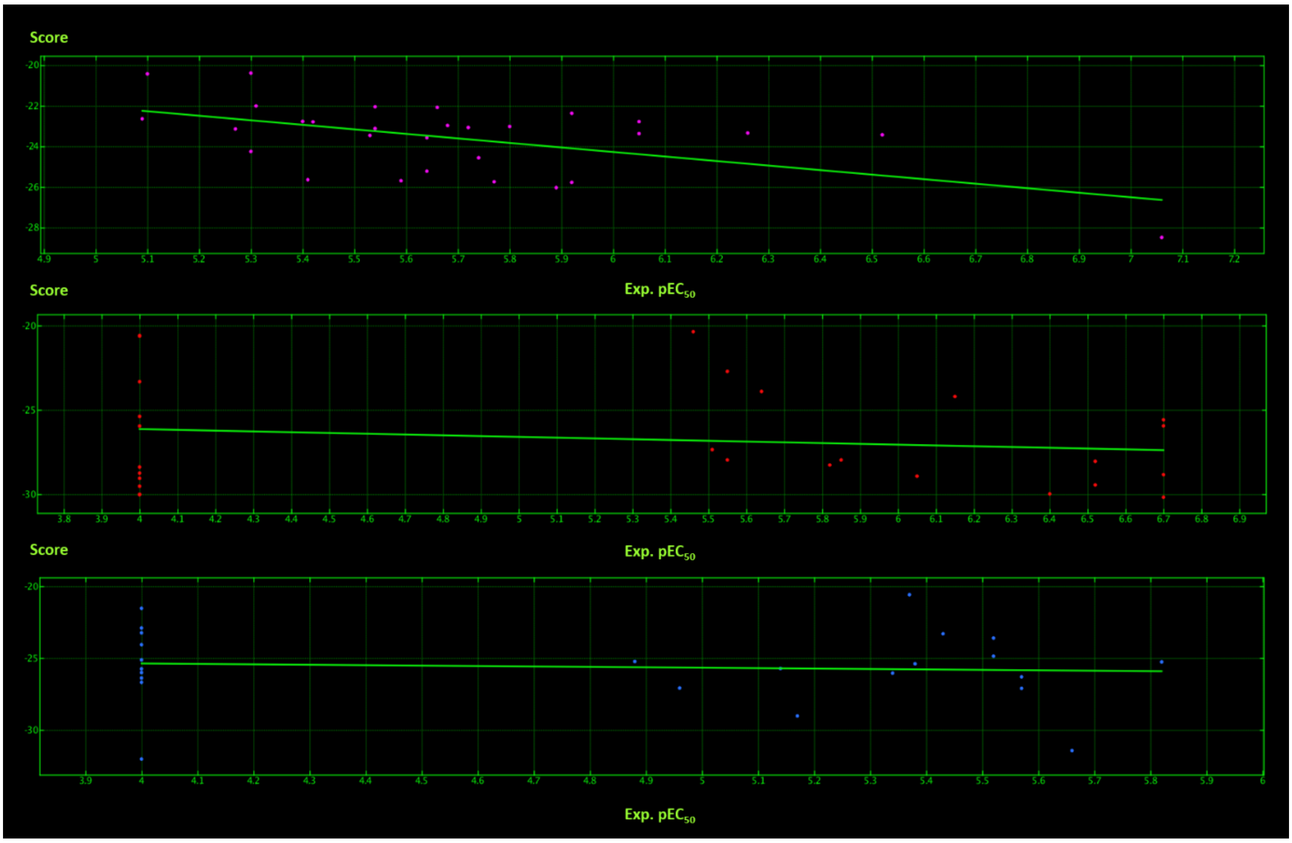

S11. Ligplot of the most relevant key contacts detected by LeadIT software concerning the docking mode of ALK-809 and SUL-809 at the modelled F508del-CFTR.

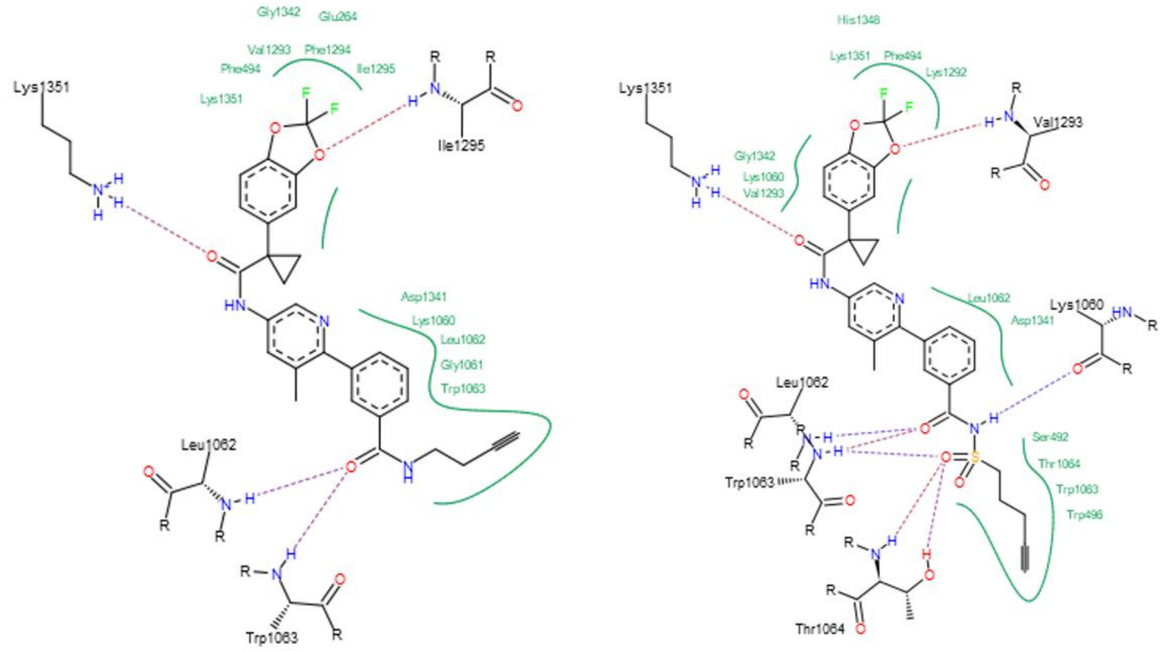

S12. Docking mode of **3** (*C* atom; dark cyan) (A) and **4** (*C* atom; pink) (B) at the *h*NBD1 domain of the F508del CFTR mutant.

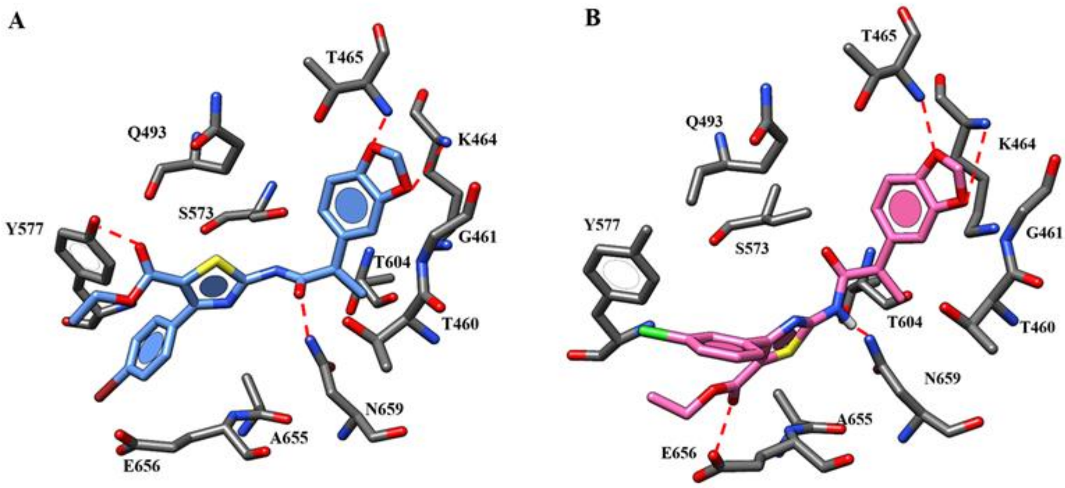

S13. Pattern of the most important interactions observed for **2**, **3**, **10** chosen as potent reference derivatives based on the substituent at the position five of the thiazole as putative NBD1-targeting correctors.

**Corrector**	**H-bonds**		**π-π stacking**	
	Amino acid residues	Ligand portion	Amino acid residues	Ligand portion
**2**	N659	Carboxamide moiety	Y577	Benzoyl moiety
	K464 T465	Benzodioxole moiety		

**3**	K464 T465	Benzodioxole moiety	Y577	p-Br-phenyl ring
	Y577	Hydroxyl group		
**4**	K464 T465	Benzodioxole moiety	Y577	m-Cl-phenyl ring
	E656	Oxygen atom of the carbonyl group		
**10**	T465 K464	Benzodioxole moiety	Y577	p-F-phenyl ring
	N659	Carboxamide group		

S14. Ligplot of the most relevant key contacts detected by LeadIT software concerning the docking mode of **3** compared to VX-809 at the modelled F508del-CFTR.

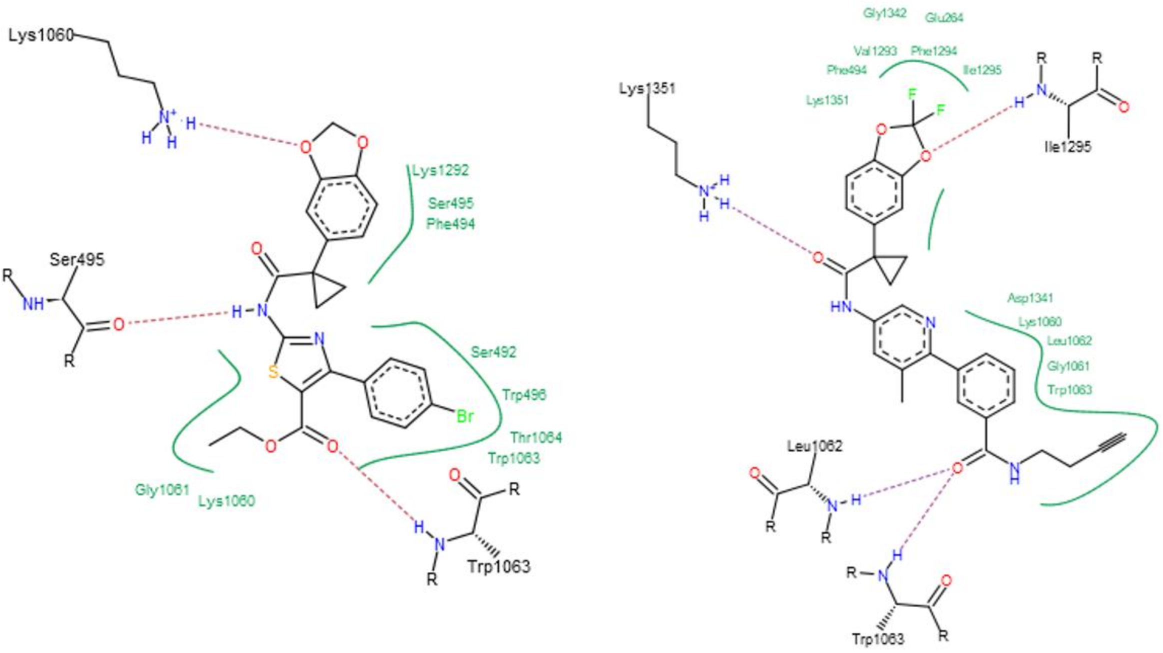

S15. Ligplot of the most relevant key contacts detected by LeadIT software concerning the docking mode of **6** compared to ALK-809 at the modelled F508del-CFTR.

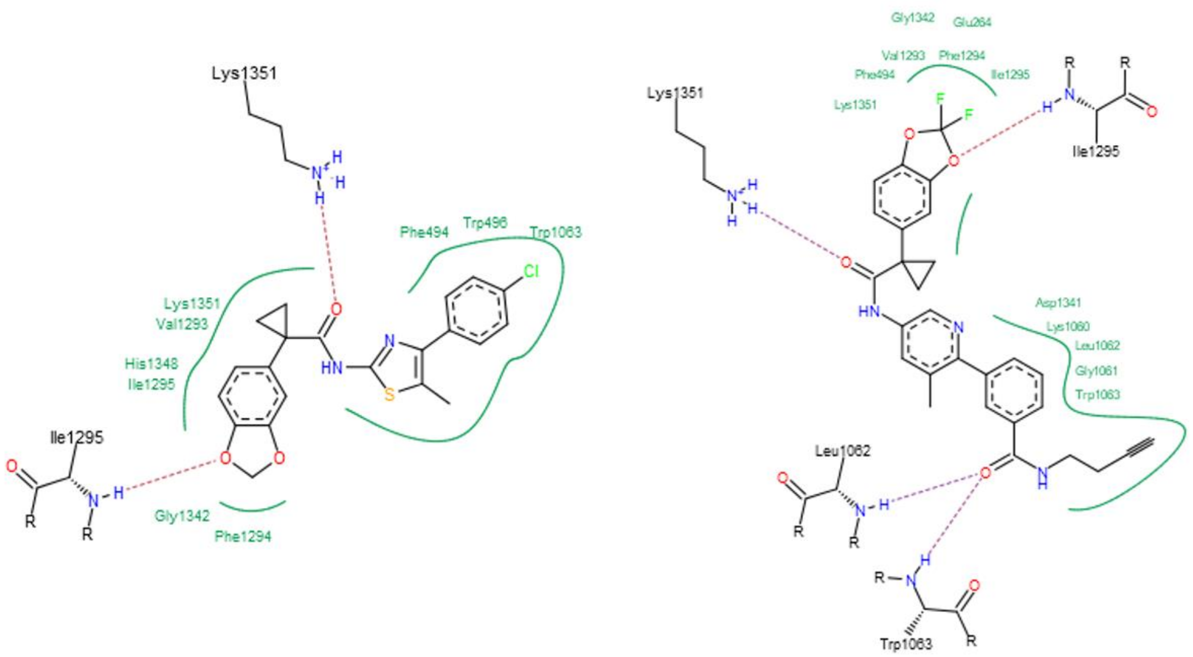

S16. Selected docking mode of **6** (*C* atom; green) and **7** (*C* atom; orange) within the modelled CFTR. The most important residues are labelled.

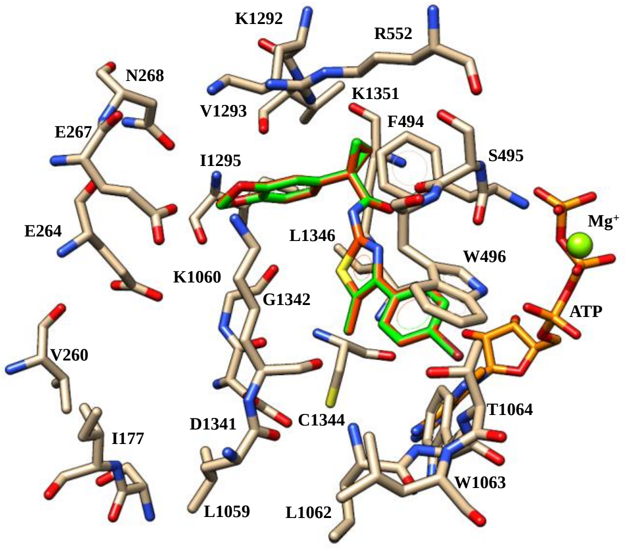

S17. Ligplot of the most relevant key contacts detected by LeadIT software concerning the docking mode of **21** compared to VX-809 at the modelled F508del-CFTR.

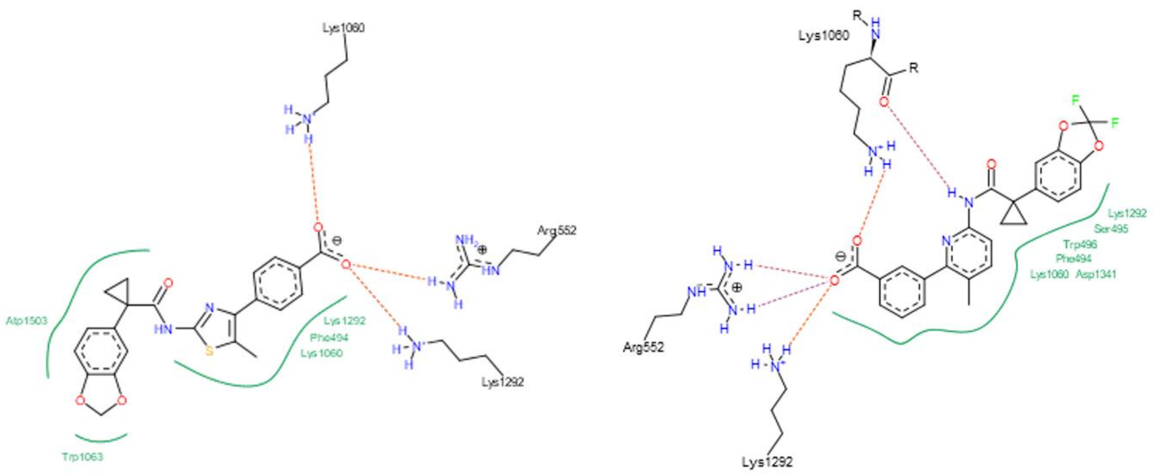

S18. Selected docking mode of **6** (*C* atom; green) and **18** (*C* atom; cyan) within the modelled CFTR mutant. The most important residues are labelled.

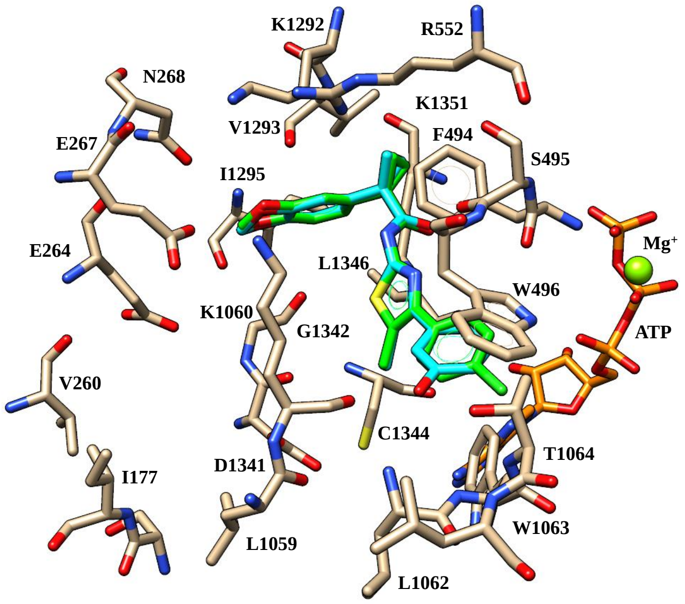

S19. Pattern of the most important interactions observed for **31**, **45**, **51** at the NBD1 domain, which have been chosen as potent reference tetrahydropyridopyrimidine correctors based on the most effective substitutions.

Corrector	H-bonds		π-π stacking	
	Amino acid residues	Ligand portion	Amino acid residues	Ligand portion
**43**	G461<br>K464	Methoxy group	F575	Benzyl moiety
	S573	Piperazine ring		
	N659	Hydroxyl group		
**46**	G461	Methoxy group	F575	Benzyl moiety

	K464			
	S573	Piperazine ring		
	T604	Hydroxyl group		
**45**	T604<br>S605	methoxy group	F575	Benzyl moiety
	N659	Imidazolyl group		
**50**	T465	Pyridine nitrogen atom	F575	4-F-benzyl moiety
	Q493	Nitrogen atom on linker		
**51**	T465	Pyridine nitrogen atom	F575	Benzyl moiety
	Q493	Nitrogen atom on linker		

S20. Docking mode of **43** (*C* atom; green) within the F508del CFTR *h*NBD1 domain. The most important residues are labelled.

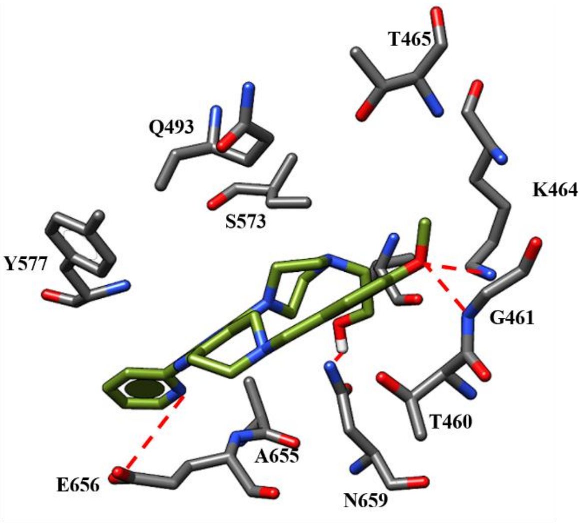

S21. Pattern of the most important interactions observed for **57**, **59**, **65**, **67** and **73** at the NBD1 domain. These compounds have been chosen as potent reference cyanoquinoline correctors based on the most effective substitutions.

Corrector	H-bonds		π-π stacking	
	Amino acid residues	Ligand portion	Amino acid residues	Ligand portion
**57**	S573<br>N659	Carboxamide linker	Y577	Quinoline core
	T465<br>G461<br>K464	Methoxy group		
**59**	N659<br>G461<br>K464	Methoxy group	Y577	Quinoline core
**65**	G461	Dimethoxy substituted phenyl ring	Y577	Quinoline core

	K464<br>T465			
	S573<br>N659	Carboxamide group		
**67**	T465	Methoxy group	Y577	Quinoline core
	S573<br>N659	Amide group		
**73**	K464<br>G461	Quinoline nitrogen atom	F575	Nicotinamide ring
	T604	Carboxamide group		

S22. Selected docking mode of **65** (C atom; blue) and **73** (C atom; dark green) within the modelled CFTR. The most important residues are labelled.

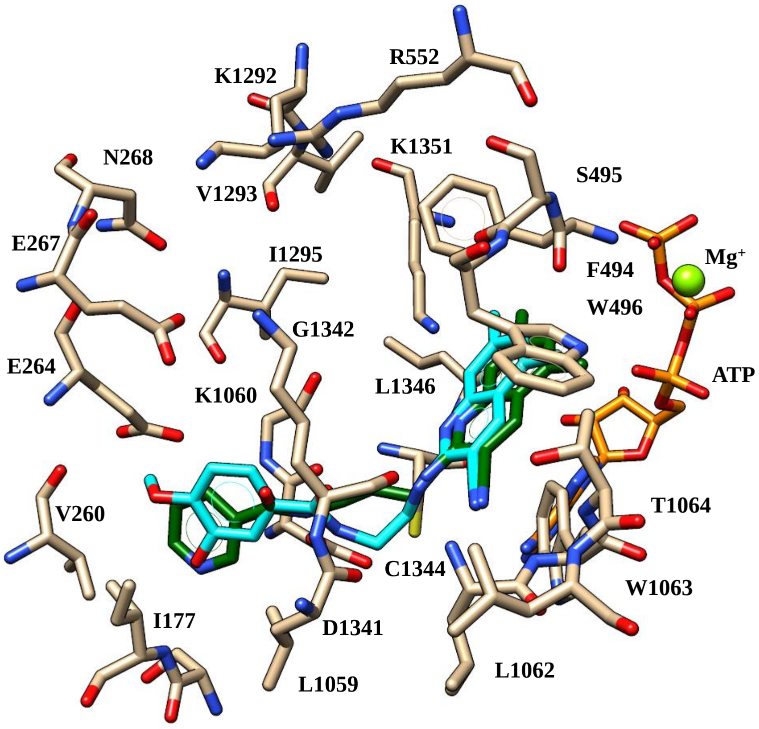

S23. Experimental (Exp.pEC_{50}) and predicted (Pred.pEC_{50}) values of the training set compounds according to the refined QSAR model A.

Cp.	Exp. pEC_{50}	E_nb	ASA-	vsa_pol	CASA+	CASA-	E_ang	Pred. pEC_{50}	Residual
**3**	6.52	40.201546	311.06015	43.506508	1127.1584	786.98218	121.59032	6.24	0.2800
**4**	6.26	40.071484	295.21408	43.506508	1153.6713	754.27197	122.66916	6.39	-0.1316
**5**	6.05	39.974373	292.46686	43.506508	1154.363	746.96039	121.56782	6.43	-0.3811
**6**	5.54	37.910526	281.6275	29.939585	792.55768	580.71594	121.07256	5.47	0.0664
**7**	5.72	38.121964	305.53513	29.939585	768.17029	622.6806	121.0268	5.20	0.5169
**8**	5.64	37.865238	282.59271	29.939585	791.71173	582.98877	121.37415	5.47	0.1736
**9**	5.80	37.621845	236.54544	29.939585	842.57831	492.01453	121.02081	5.92	-0.1225
**10**	6.05	36.758995	252.77013	29.939585	846.78784	542.69745	122.49203	6.01	0.0352
**11**	5.54	37.211922	277.50491	29.939585	728.87549	562.22498	120.49342	5.35	0.1880
**12**	5.31	37.436359	298.69312	29.939585	708.33789	597.98364	120.47495	5.10	0.2088
**13**	5.09	37.212086	280.57724	29.939585	743.19354	569.01068	120.71957	5.37	-0.2798
**15**	5.68	35.795937	244.57336	29.939585	795.67621	516.53894	122.20341	5.98	-0.2962
**16**	5.74	37.433041	230.66957	43.506508	890.95435	525.00391	121.43837	5.75	-0.0125
**17**	5.64	37.343327	234.04518	43.506508	893.51337	533.62299	121.48071	5.76	-0.1192
**18**	5.92	38.131062	238.4789	43.506508	966.19189	552.31714	121.81725	5.90	0.0249
**23**	5.42	35.323212	381.84894	29.939585	698.24213	844.64984	120.17078	5.32	0.0989
**24**	5.27	35.113384	358.79428	29.939585	723.77502	802.6228	120.48874	5.57	-0.3021

**25**	5.89	34.822216	308.6485	43.506508	804.03973	757.4234	120.57159	5.84	0.0467
**26**	5.77	34.670029	308.59375	43.506508	799.9693	756.05463	120.56307	5.84	-0.0710
**27**	5.40	34.575485	355.2095	29.939585	662.57629	782.17133	119.65387	5.42	-0.0218
**30**	5.55	82.923752	171.84309	30.614649	1648.9868	370.83737	20.736029	5.68	-0.1298
**31**	5.85	82.42186	184.62444	48.35714	1847.3866	444.39102	22.800692	5.61	0.2405
**32**	5.55	106.46642	171.46677	48.35714	2676.562	455.58719	27.980944	5.44	0.1107
**37**	4.00	144.48102	122.29909	22.730305	3444.0505	287.52515	32.371471	4.37	-0.3663
**38**	4.00	153.96991	127.36039	17.047728	3526.8616	297.38651	36.52422	3.73	0.2738
**39**	4.00	147.62102	126.22911	22.730305	3584.5537	293.60892	32.575783	4.36	-0.3587
**40**	4.00	154.23276	123.18449	17.047728	3532.3589	284.55618	35.389881	3.73	0.2680
**41**	4.00	158.429	119.46897	19.551485	4025.5757	309.06622	38.373356	4.62	-0.6234
**42**	4.00	153.73146	153.67113	17.047728	3514.4336	374.03552	36.782146	3.71	0.2902
**43**	6.40	110.90891	133.17619	33.118404	3017.5261	351.71832	33.262741	6.52	-0.1196
**44**	5.46	87.505989	170.54222	50.860893	2184.323	448.35553	26.178873	6.06	-0.5979
**45**	6.70	84.29599	134.07512	30.916637	2102.2649	341.48932	57.236408	6.49	0.2082
**46**	5.64	111.44763	156.84193	50.860893	3098.0825	451.54794	31.014784	6.08	-0.4432
**47**	6.52	93.705719	173.39532	33.118404	2326.9932	451.52142	33.383991	6.42	0.0951
**49**	6.15	86.606331	225.05911	36.297226	1873.995	530.46436	21.122585	5.62	0.5286
**50**	6.70	74.338272	152.44916	41.979801	1729.2054	368.77454	21.081335	6.62	0.0827
**54**	6.05	148.64999	98.055092	17.047728	3884.696	228.27225	34.709503	5.37	0.6825
**55**	4.00	132.28711	164.27565	30.614649	2949.2405	432.04498	31.816795	4.15	-0.1528
**56**	4.00	123.07984	190.38725	49.064251	2928.6514	488.53369	46.862576	3.77	0.2313
**57**	5.66	55.075397	237.48724	50.860893	923.78021	500.8606	22.470055	5.35	0.3140
**58**	4.96	58.179813	234.20871	50.860893	911.40234	502.84613	22.384676	5.06	-0.1031
**59**	5.52	56.934811	234.43777	50.860893	922.17236	493.72595	22.341722	5.16	0.3606
**60**	4.00	54.783257	230.61339	54.039715	789.38843	457.07571	20.474987	4.76	-0.7638
**61**	5.57	51.930538	229.52635	54.039715	774.53314	448.72403	19.566298	5.01	0.5581
**62**	5.14	50.999371	214.56854	54.039715	781.28369	413.47357	20.403036	5.17	-0.0281
**63**	4.00	51.904221	252.33662	48.35714	709.98688	475.40222	19.385567	4.85	-0.8527
**65**	5.82	63.475273	226.01973	53.364651	1141.8444	538.83099	24.826355	5.33	0.4917
**66**	5.17	57.026089	191.64943	59.72229	882.32562	403.23041	19.090767	4.93	0.2428
**67**	5.57	60.584358	218.01747	56.543472	1009.9583	490.53931	24.855558	4.98	0.5911
**68**	5.43	62.59671	226.41823	53.364651	1151.2058	546.57361	23.791538	5.51	-0.0800
**70**	5.34	58.139511	219.83636	50.860893	882.26208	469.1308	15.243285	5.19	0.1472
**71**	5.37	57.90588	214.09308	50.860893	880.84534	456.23233	15.861175	5.23	0.1426
**73**	5.52	54.521137	204.4958	54.039715	790.16455	404.9017	12.876506	5.11	0.4106
**74**	4.88	52.395954	204.63695	54.039715	747.9267	399.45132	13.675772	5.17	-0.2899
**75**	4.00	71.220154	226.46532	39.495743	989.84216	478.97415	20.628365	4.45	-0.4482
**76**	4.00	71.40937	230.99619	39.495743	977.70166	479.77908	22.155819	4.28	-0.2833
**77**	4.00	71.750427	227.05901	39.495743	986.42834	470.92038	22.093498	4.29	-0.2889
**78**	4.00	70.656143	224.05431	42.674564	843.64532	437.12994	19.305321	3.78	0.2229
**79**	4.00	66.774406	216.00131	42.674564	835.51776	415.37051	20.192486	4.16	-0.1615

**80**	4.00	65.905418	207.23102	42.674564	843.74304	392.70279	19.777443	4.30	-0.3036

S24. Experimental (Exp.pEC$_{50}$) and predicted (Pred.pEC$_{50}$) values of the test set compounds according to the refined QSAR model A.

**Cp.**	**Exp. pEC$_{50}$**	**E_nb**	**ASA-**	**vsa_pol**	**CASA+**	**CASA-**	**E_ang**	**Pred. pEC$_{50}$**	**Residual**
**1**	5.59	48.448597	381.67267	57.073425	725.31787	1243.8712	108.23756	5.93	-0.3400
**2**	7.06	53.35915	305.88519	43.506508	1120.5023	774.80719	121.30415	6.65	0.4051
**14**	5.92	37.931583	304.58237	29.939585	707.73633	608.5556	120.34983	5.41	0.5145
**19**	5.41	38.162426	238.59331	43.506508	953.86444	551.62775	121.88371	5.85	-0.4416
**20**	5.30	39.625359	255.47565	57.073425	1019.7084	648.90814	19.199339	5.63	-0.3321
**21**	5.53	40.260384	255.33957	57.073425	1096.9369	657.75476	19.016466	5.83	-0.3000
**22**	5.30	40.724495	233.93356	49.189083	1171.973	592.08582	18.133121	5.65	-0.3458
**28**	5.10	36.033928	246.28993	24.25701	585.13702	430.02222	106.14865	4.32	0.7800
**29**	5.66	48.590977	206.37631	29.939585	706.36316	397.68716	107.79346	5.34	0.3218
**33**	5.51	146.15053	133.94344	22.730305	3419.7056	318.91934	30.880795	5.14	0.3719
**34**	5.82	153.23914	136.87323	17.047728	3544.1233	323.7052	35.1539	5.87	-0.0456
**35**	4.00	82.230225	180.92363	48.35714	1837.0845	429.87457	25.189426	4.33	-0.3304
**36**	4.00	106.19363	165.49545	48.35714	2657.3655	434.75656	29.022572	4.38	-0.3754
**48**	4.00	89.662857	233.99181	30.614649	1898.8805	553.62463	30.238689	4.97	-0.9700
**51**	6.70	98.400475	154.97318	22.730305	2273.0305	328.85309	26.189623	5.97	0.7289
**52**	6.70	138.94405	110.62077	17.047728	3588.1072	261.39685	55.438435	6.18	0.5214
**53**	6.52	158.92085	142.09158	17.047728	3969.3804	352.67133	36.833698	6.42	0.0959
**64**	5.38	63.195755	223.05199	53.364651	1137.3612	529.30237	24.910639	5.64	-0.2592
**69**	4.00	61.174698	178.73625	50.860893	932.14954	388.21515	14.915898	4.27	-0.2692
**72**	4.00	54.135616	218.24225	54.039715	736.79474	438.01221	13.264742	3.96	0.0440

S25. PCA score plot computed on the data matrix of molecular descriptors and model response. The training set and the test set compounds selected by the Kennard Stone algorithm are represented in black and in red, respectively.

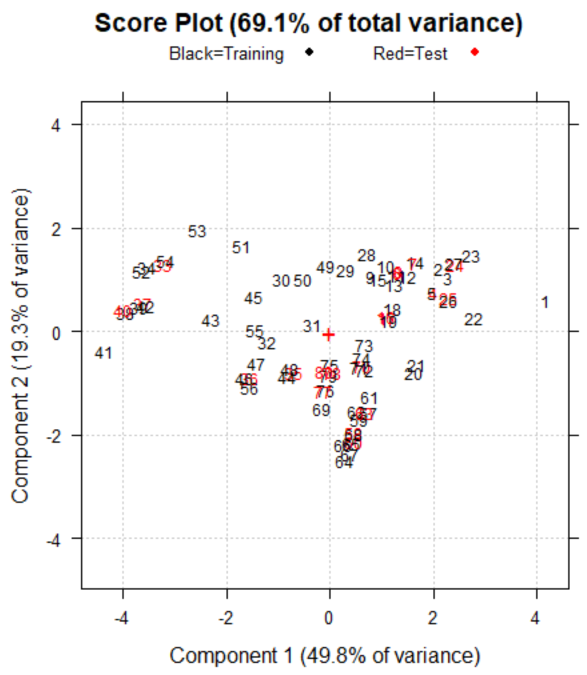

S26. Experimental (Exp.pEC50) and predicted (Pred.pEC50) values of the training set compounds according to the refined QSAR model B.

Cp.	Exp. pEC_{50}	E_nb	ASA-	vsa_pol	CASA+	CASA-	E_ang	Pred. pEC_{50}	Residual
**1**	5.59	48.448597	381.67267	57.073425	725.31787	1243.8712	108.23756	5.61	-0.0209
**2**	7.06	53.35915	305.88519	43.506508	1120.5023	774.80719	121.30415	5.25	1.8127
**3**	6.52	40.201546	311.06015	43.506508	1127.1584	786.98218	121.59032	6.35	0.1692
**5**	6.05	39.974373	292.46686	43.506508	1154.363	746.96039	121.56782	6.51	-0.4591
**9**	5.80	37.621845	236.54544	29.939585	842.57831	492.01453	121.02081	5.81	-0.0101
**10**	6.05	36.758995	252.77013	29.939585	846.78784	542.69745	122.49203	6.15	-0.0956
**11**	5.54	37.211922	277.50491	29.939585	728.87549	562.22498	120.49342	5.60	-0.0568
**12**	5.31	37.436359	298.69312	29.939585	708.33789	597.98364	120.47495	5.40	-0.0854
**13**	5.09	37.212086	280.57724	29.939585	743.19354	569.01068	120.71957	5.61	-0.5246

**14**	5.92	37.931583	304.58237	29.939585	707.73633	608.5556	120.34983	5.32	0.6010
**15**	5.68	35.795937	244.57336	29.939585	795.67621	516.53894	122.20341	6.11	-0.4272
**18**	5.92	38.131062	238.4789	43.506508	966.19189	552.31714	121.81725	6.08	-0.1582
**19**	5.41	38.162426	238.59331	43.506508	953.86444	551.62775	121.88371	6.04	-0.6319
**20**	5.30	39.6254	255.4757	57.0734	1.019.7084	648.9081	19.1993	5.35	-0.0491
**21**	5.53	40.2604	255.3396	57.0734	1.096.9369	657.7548	19.0165	5.56	-0.0300
**22**	5.30	40.7245	233.9336	49.1891	1.171.9730	592.0858	18.1331	4.97	0.3346
**23**	5.42	35.323212	381.84894	29.939585	698.24213	844.64984	120.17078	5.54	-0.1183
**26**	5.77	34.670029	308.59375	43.506508	799.9693	756.05463	120.56307	5.97	-0.2045
**27**	5.40	34.575485	355.2095	29.939585	662.57629	782.17133	119.65387	5.61	-0.2125
**28**	5.10	36.033928	246.28993	24.25701	585.13702	430.02222	106.14865	5.55	-0.4541
**29**	5.66	48.590977	206.37631	29.939585	706.36316	397.68716	107.79346	4.98	0.6847
**30**	5.55	82.923752	171.84309	30.614649	1648.9868	370.83737	20.736029	5.58	-0.0263
**31**	5.85	82.42186	184.62444	48.35714	1847.3866	444.39102	22.800692	5.58	0.2712
**32**	5.55	106.46642	171.46677	48.35714	2676.562	455.58719	27.980944	5.61	-0.0648
**34**	5.82	153.23914	136.87323	17.047728	3544.1233	323.7052	35.1539	4.54	1.2820
**38**	4.00	153.96991	127.36039	17.047728	3526.8616	297.38651	36.52422	4.43	-0.4326
**39**	4.00	147.62102	126.22911	22.730305	3584.5537	293.60892	32.575783	4.94	-0.9381
**41**	4.00	158.429	119.46897	19.551485	4025.5757	309.06622	38.373356	5.25	-1.2455
**42**	4.00	153.73146	153.67113	17.047728	3514.4336	374.03552	36.782146	4.41	-0.4097
**43**	6.40	110.90891	133.17619	33.118404	3017.5261	351.71832	33.262741	6.55	-0.1504

**44**	5.46	87.505989	170.54222	50.860893	2184.323	448.35553	26.178873	6.01	-0.5489
**45**	6.70	84.29599	134.07512	30.916637	2102.2649	341.48932	57.236408	6.46	0.2391
**46**	5.64	111.44763	156.84193	50.860893	3098.0825	451.54794	31.014784	6.22	-0.5776
**47**	6.52	93.705719	173.39532	33.118404	2326.9932	451.52142	33.383991	6.33	0.1853
**48**	4.00	89.662857	233.99181	30.614649	1898.8805	553.62463	30.238689	5.44	-1.4435
**49**	6.15	86.606331	225.05911	36.297226	1873.995	530.46436	21.122585	5.57	0.5850
**50**	6.70	74.338272	152.44916	41.979801	1729.2054	368.77454	21.081335	6.34	0.3561
**51**	6.70	98.400475	154.97318	22.730305	2273.0305	328.85309	26.189623	5.94	0.7553
**52**	6.70	138.94405	110.62077	17.047728	3588.1072	261.39685	55.438435	5.69	1.0133
**53**	6.52	158.92085	142.09158	17.047728	3969.3804	352.67133	36.833698	5.07	1.4543
**54**	6.05	148.64999	98.055092	17.047728	3884.696	228.27225	34.709503	5.80	0.2471
**55**	4.00	132.28711	164.27565	30.614649	2949.2405	432.04498	31.816795	4.64	-0.6378
**56**	4.00	123.07984	190.38725	49.064251	2928.6514	488.53369	46.862576	4.42	-0.4237
**57**	5.66	55.075397	237.48724	50.860893	923.78021	500.8606	22.470055	5.21	0.4507
**59**	5.52	56.934811	234.43777	50.860893	922.17236	493.72595	22.341722	5.06	0.4595
**61**	5.57	51.930538	229.52635	54.039715	774.53314	448.72403	19.566298	4.92	0.6509
**62**	5.14	50.999371	214.56854	54.039715	781.28369	413.47357	20.403036	5.05	0.0896
**64**	5.38	63.195755	223.05199	53.364651	1137.3612	529.30237	24.910639	5.24	0.1396
**65**	5.82	63.475273	226.01973	53.364651	1141.8444	538.83099	24.826355	5.23	0.5890
**66**	5.17	57.026089	191.64943	59.72229	882.32562	403.23041	19.090767	4.87	0.3011
**67**	5.57	60.584358	218.01747	56.543472	1009.9583	490.53931	24.855558	4.94	0.6261

**68**	5.43	62.59671	226.41823	53.364651	1151.2058	546.57361	23.791538	5.37	0.0599
**69**	4.00	61.174698	178.73625	50.860893	932.14954	388.21515	14.915898	5.11	-1.1142
**71**	5.37	57.90588	214.09308	50.860893	880.84534	456.23233	15.861175	5.08	0.2940
**72**	4.00	54.135616	218.24225	54.039715	736.79474	438.01221	13.264742	4.83	-0.8345
**73**	5.52	54.521137	204.4958	54.039715	790.16455	404.9017	12.876506	4.97	0.5528
**74**	4.88	52.395954	204.63695	54.039715	747.9267	399.45132	13.675772	5.01	-0.1328
**75**	4.00	71.220154	226.46532	39.495743	989.84216	478.97415	20.628365	4.48	-0.4822
**76**	4.00	71.40937	230.99619	39.495743	977.70166	479.77908	22.155819	4.36	-0.3586
**79**	4.00	66.774406	216.00131	42.674564	835.51776	415.37051	20.192486	4.24	-0.2422

S27. Experimental (Exp.pEC$_{50}$) and predicted (Pred.pEC$_{50}$) values of the test set compounds according to the refined QSAR model B.

**Cp.**	**Exp. pEC$_{50}$**	**E_nb**	**ASA-**	**vsa_pol**	**CASA+**	**CASA-**	**E_ang**	**Pred. pEC$_{50}$**	**Residual**
**4**	6.26	40.071484	295.21408	43.506508	1153.6713	754.27197	122.66916	6.48	-0.2219
**6**	5.54	37.910526	281.6275	29.939585	792.55768	580.71594	121.07256	5.70	-0.1649
**7**	5.72	38.121964	305.53513	29.939585	768.17029	622.6806	121.0268	5.49	0.2333
**8**	5.64	37.865238	282.59271	29.939585	791.71173	582.98877	121.37415	5.70	-0.0602
**16**	5.74	37.433041	230.66957	43.506508	890.95435	525.00391	121.43837	5.95	-0.2126
**17**	5.64	37.343327	234.04518	43.506508	893.51337	533.62299	121.48071	5.96	-0.3180
**24**	5.27	35.113384	358.79428	29.939585	723.77502	802.6228	120.48874	5.74	-0.4730
**25**	5.89	34.822216	308.6485	43.506508	804.03973	757.4234	120.57159	5.98	-0.0867
**33**	5.51	146.15053	133.94344	22.730305	3419.7056	318.91934	30.880795	4.72	0.7905
**35**	4.00	82.230225	180.92363	48.35714	1837.0845	429.87457	25.189426	4.53	-0.5273
**36**	4.00	106.19363	165.49545	48.35714	2657.3655	434.75656	29.022572	5.17	-1.1682
**37**	4.00	144.48102	122.29909	22.730305	3444.0505	287.52515	32.371471	4.92	-0.9154
**40**	4.00	154.23276	123.18449	17.047728	3532.3589	284.55618	35.389881	4.43	-0.4347
**58**	4.96	58.179813	234.20871	50.860893	911.40234	502.84613	22.384676	4.98	-0.0186
**60**	4.00	54.783257	230.61339	54.039715	789.38843	457.07571	20.474987	4.73	-0.7267
**63**	4.00	51.904221	252.33662	48.35714	709.98688	475.40222	19.385567	4.78	-0.7768

**70**	5.34	58.139511	219.83636	50.860893	882.26208	469.1308	15.243285	5.05	0.2946
**77**	4.00	71.750427	227.05901	39.495743	986.42834	470.92038	22.093498	4.36	-0.3648
**78**	4.00	70.656143	224.05431	42.674564	843.64532	437.12994	19.305321	3.93	0.0680
**80**	4.00	65.905418	207.23102	42.674564	843.74304	392.70279	19.777443	4.36	-0.3568

S28. Distribution of the experimental (Exp.pEC50) potency values of the test set compounds with respect to the E_nb and CASA+ (Å²) descriptors. All the test set correctors are reported as magenta crosses.

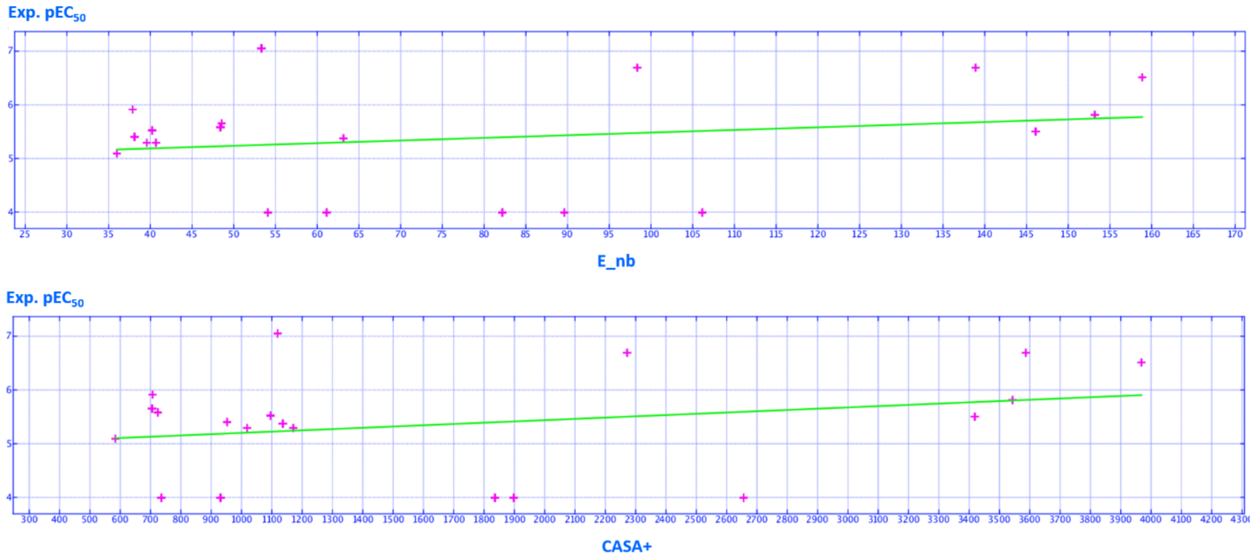

S29. Distribution of the experimental (Exp.pEC50) potency values of the hybrids included in the training set with respect to the E_ang (Kcal/mol). ASA- (Å²) and CASA- (Å²). descriptors. Hybrid derivatives are reported as cyan spheres.

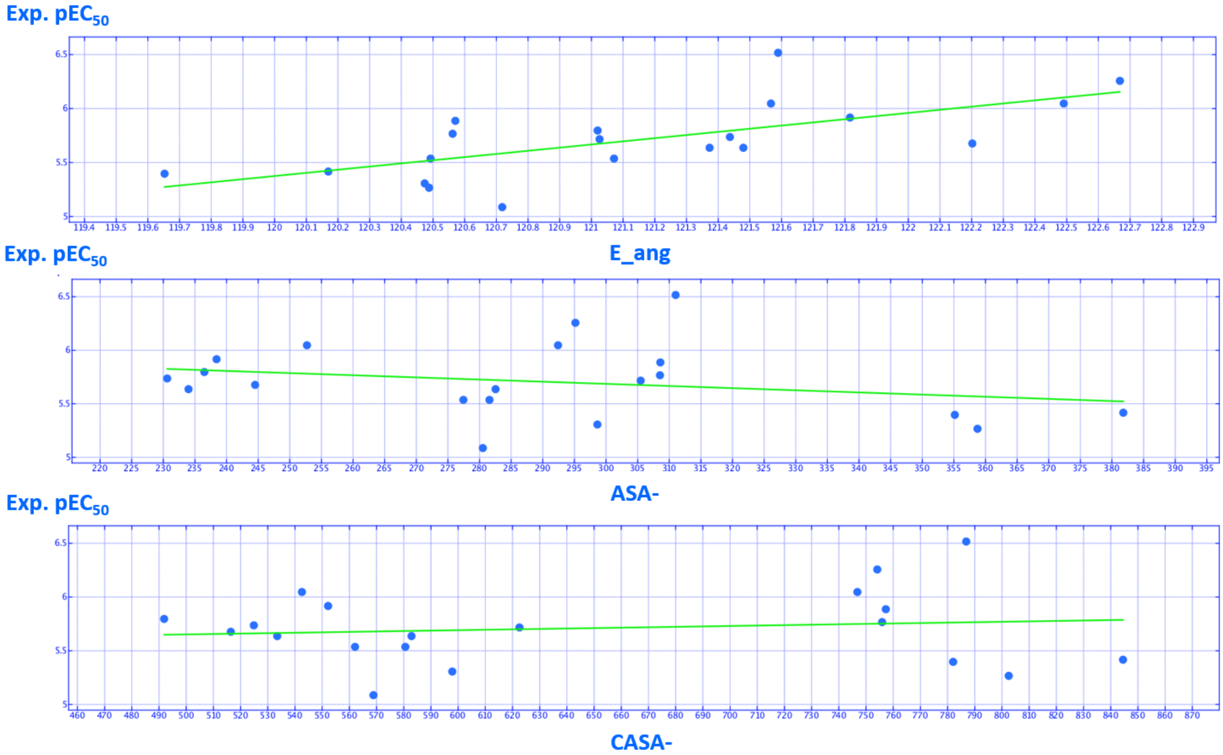

S30. Distribution of the experimental (Exp.pEC50) potency values of the tetrahydropyridopyrimidines included in the training set with respect to the ASA- (Å^2) and CASA- (Å^2) and vsa_pol descriptors. Tetrahydropyridopyrimidine derivatives are reported as orange spheres.

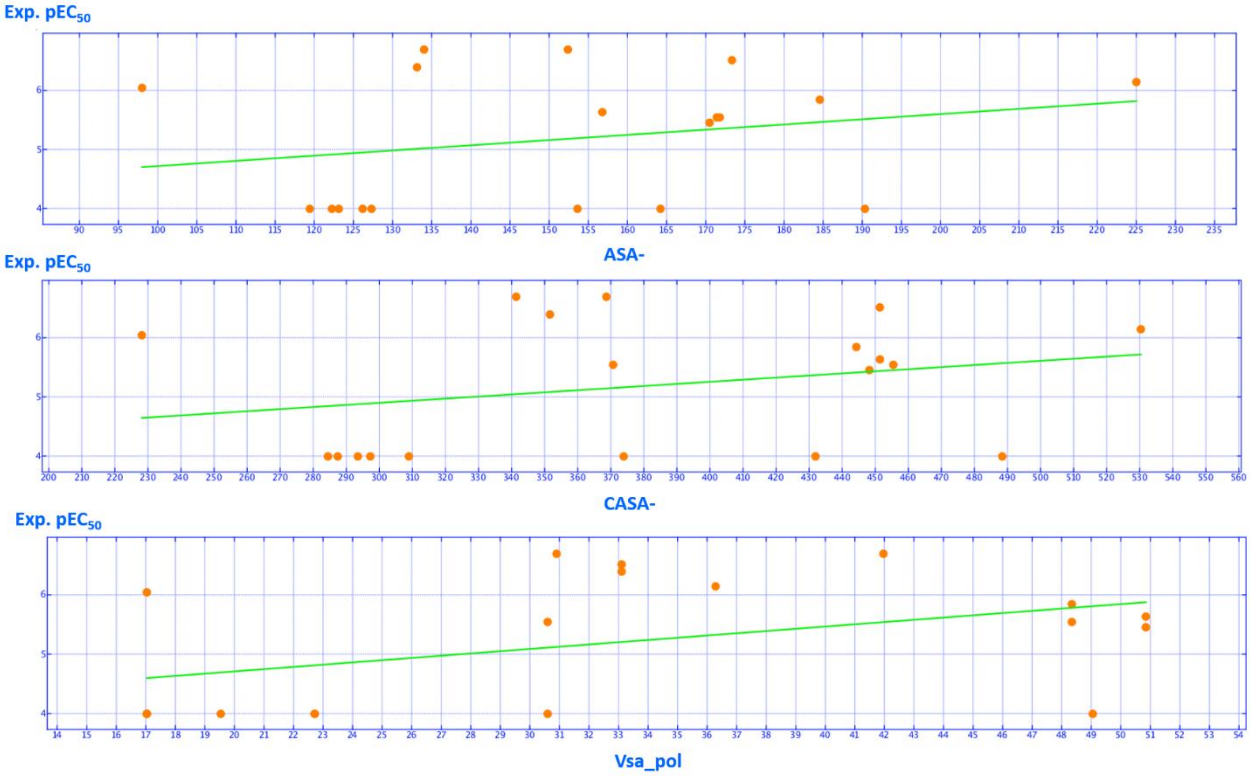

Supplement: Supplementary file 1 [file ijms-21-08084-s001.pdf]
